# Surface-induced enantiomorphic crystallization of achiral fullerene derivatives in thin films†

**DOI:** 10.1039/d0sc01163k

**Published:** 2020-04-17

**Authors:** Chao Wang, Hua Hao, Daisuke Hashizume, Keisuke Tajima

**Affiliations:** RIKEN Center for Emergent Matter Science (CEMS) 2-1 Hirosawa, Wako Saitama 351-0198 Japan keisuke.tajima@riken.jp

## Abstract

The chirality of organic semiconductors is important for various applications in optoelectronics and spintronics. Here, we propose a new strategy to induce structural chirality in achiral organic semiconductors in thin films. Enantiomeric fullerene derivatives (*S*)-pSi and (*R*)-pSi, which have oligo(dimethylsiloxane) as a low-surface-energy moiety, were synthesized and used as surface-segregated monolayers (SSMs) in spin-coated films of several achiral fullerene derivatives. Upon thermal annealing, the presence of the chiral SSMs led to the crystallization of the fullerenes in the films as an SSM-induced crystal phase at lower temperatures. The crystallized films showed circular dichroism ascribed to the fullerene absorption, the sign and the intensity of which depended on the handedness of the SSM molecules and the film thickness, respectively. These results indicate that the achiral fullerene derivatives in the films were induced by the SSMs to crystallize into enantiomorphic crystals. Our approach to inducing chirality in organic thin films is compatible with many device applications.

## Abstract 2

Chiral induction: surface-segregated monolayers of chiral molecules induce the enantiomorphic crystallization of achiral fullerene derivatives in thin films.

## Graphical abstract

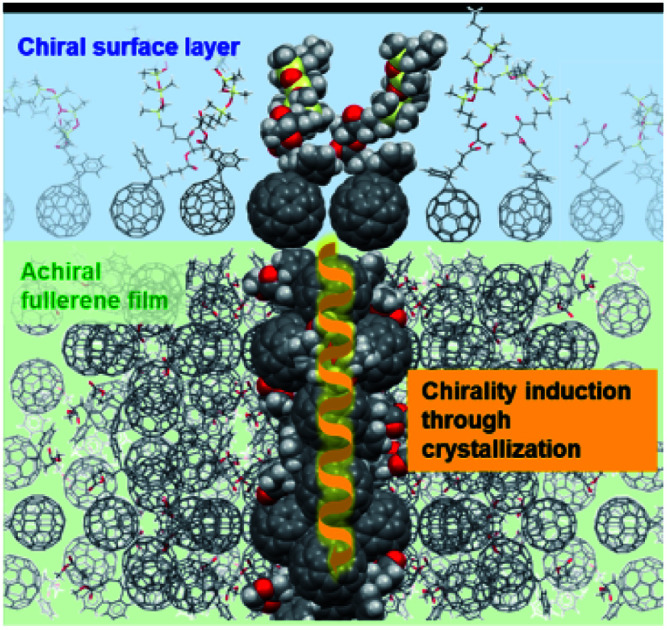

## Introduction

Chirality is a basic property in nature and is observed across all length scales, from elemental particles to macroscopic objects.^1^ Chiral molecules with left- and right-handed chemical structures linked by mirror symmetry (enantiomers) are of fundamental importance in the life sciences and medicine.^2–5^ In addition, in assemblies of molecules, the symmetry of the packing structure is the key to expressing the chirality of bulk materials as a whole and enantiomeric structures can even be formed from achiral molecules. Chiral materials are useful for advanced applications in nonlinear optics,^6,7^ stereospecific chemistry,^8–11^ and spintronics^12–15^ owing to their interactions with electromagnetic waves, molecules, and electronic spin, respectively. For example, chiral films can be used in optoelectronic devices that emit or detect circularly polarized light.^16^

Chiral induction, where an assembly of achiral species acquires structural chirality by interacting with a chiral species, provides a way to create chirality in condensed matter.^17,18^ Chiral induction has been reported in self-assembled structures in solution,^19–21^ crystallization from solution,^22–25^ and liquid crystals.^26–29^ For thin film form of the materials which is relevant to the applications, chiral dopants can induce structural chirality in films of achiral semiconducting polymers.^30–32^ However, the mixing of the dopants affects the film structure and interferes with the properties of the organic semiconductors. The application of chiral stimuli to the materials, such as irradiation with circularly polarized light, can also induce enantiomorphic structures,^33–35^ but the enantiomorphic effects are generally weak. To our knowledge, chiral induction during crystallization has not been reported for thin-film organic semiconductors.

**Fig. 1:**
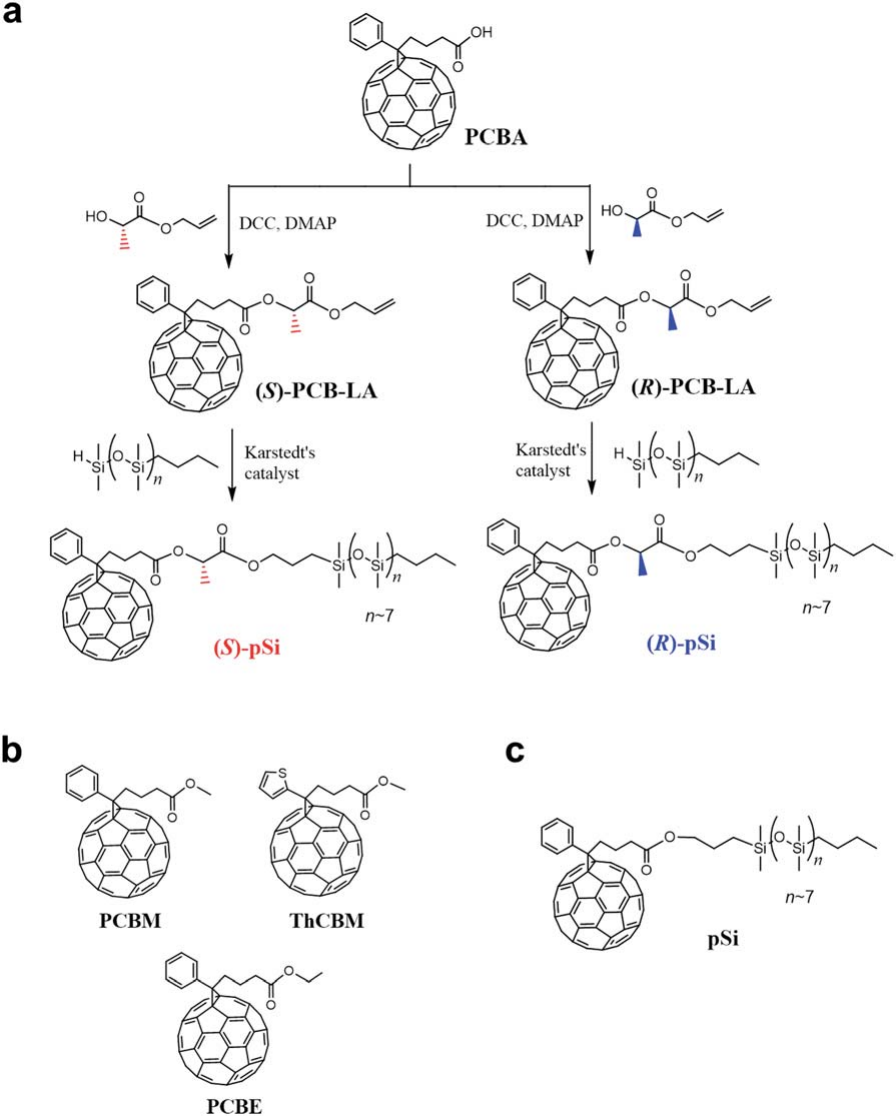
(a) Synthetic routes for (*S*)-pSi and (*R*)-pSi. Chemical structures of (b) the achiral fullerene derivatives used for the inside of the films, PCBM, ThCBM, and PCBE, and (c) pSi.

Thin-film organic semiconductors with high structural order are critical components in organic electronic devices. We have been developing surface-segregated monolayers (SSMs) for modifying the surface of organic semiconductor films and controlling film structures.^36–42^ SSMs can be prepared using a blend solution of a base organic semiconductor and a surface modifier that consists of a semiconducting part and a moiety with low surface energy, such as fluoroalkyl or oligosiloxane chains. This molecular design drives spontaneous segregation of the modifiers to the surface as a monolayer with a preferred molecular alignment during coating to minimize the total energy of the system. The phenomeon can be understood in an analogous way to the Langmuir adsorption of surfactants at liquid/air interfaces.^43^ Recently, we discovered that SSMs based on a fullerene derivative (*e.g.*, pSi in Fig. 1c) induce the crystallization of [6,6]-phenyl-C_61_-butyric acid methyl ester (PCBM) inside the film from the surface to give PCBM films with an unprecedented high crystallinity after thermal annealing.^42^ Structural analysis revealed that the crystal structure of PCBM was induced by the SSMs. Importantly, the SSM-induced crystal structure in the PCBM film belonged to a non-centrosymmetric space group and the unique axis was aligned in the vertical direction of the films, reflecting the directed crystal growth from the surface. These findings led us to the hypothesis that crystalline PCBM films can acquire structural chirality through crystallization from the surface.

**Fig. 2:**
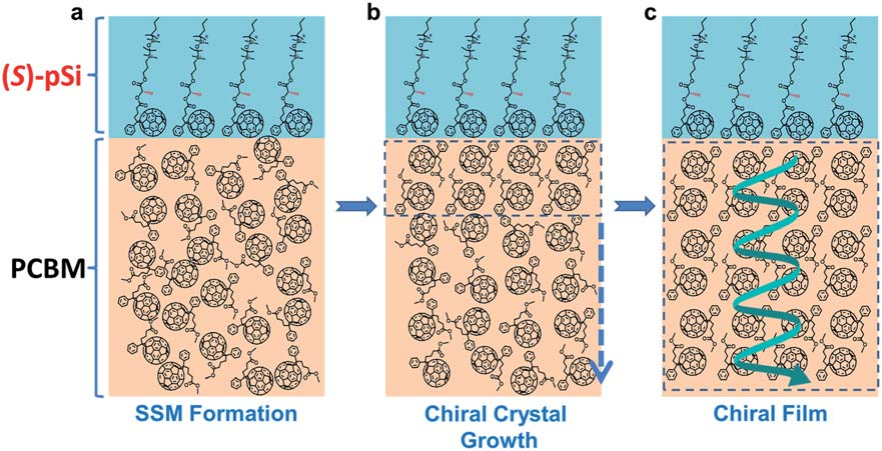
Schematics of chiral induction in the (*S*)-pSi/PCBM system. (a) In as-cast films of (*S*)-pSi/PCBM, the (*S*)-pSi SSM forms with the preferential molecular orientation, and (b) during thermal annealing, the (*S*)-pSi SSM induces the crystallization of PCBM inside the film with a single-handed helicity along the vertical direction to form (c) the crystallized PCBM films with the enantiomorphic structure after thermal annealing.

In this study, we synthesized new chiral surface modifiers: (*S*)-pSi and (*R*)-pSi (Fig. 1a). Lactic acid was used as the chiral building block to connect the fullerene part and the oligosiloxane moiety with low surface energy. Both the compounds were expected to function as surface modifiers to form SSMs in the PCBM films, as in the case of pSi/PCBM. The crystalline materials inside the films were investigated for other achiral fullerene derivatives with higher crystallinity ([6,6]-thienyl-C_61_-butyric acid methyl ester (ThCBM) and [6,6]-phenyl-C_61_ butyric acid ethyl ester (PCBE), Fig. 1b). We hypothesized that the (*S*)-pSi and (*R*)-pSi SSMs induce the chirality of the crystal packing structure of the achiral fullerene derivatives inside the films through surface-induced crystallization (Fig. 2).

## Results and discussion

### Synthesis

Fig. 1a shows the synthetic routes for chiral surface modifiers (*S*)-pSi and (*R*)-pSi. Precursor (*S*)-PCB-LA was synthesized by the Steglich esterification reaction between [6,6]-phenyl-C_61_-butyric acid (PCBA) and allyl l-(−)-lactate in the presence of *N*,*N*′-dicyclohexylcarbodiimide (DCC) and 4-dimethylaminopyridine (DMAP), while the esterification between PCBA and allyl d-(+)-lactate gave (*R*)-PCB-LA. Chiral surface modifiers (*S*)-pSi and (*R*)-pSi were prepared by the hydrosilylation of (*S*)-PCB-LA and (*R*)-PCB-LA, respectively, using *n*-butyl/hydride-terminated polydimethylsiloxane. The detail synthesis and characterization are described in the ESI.†

### Formation of SSM

**Fig. 3:**
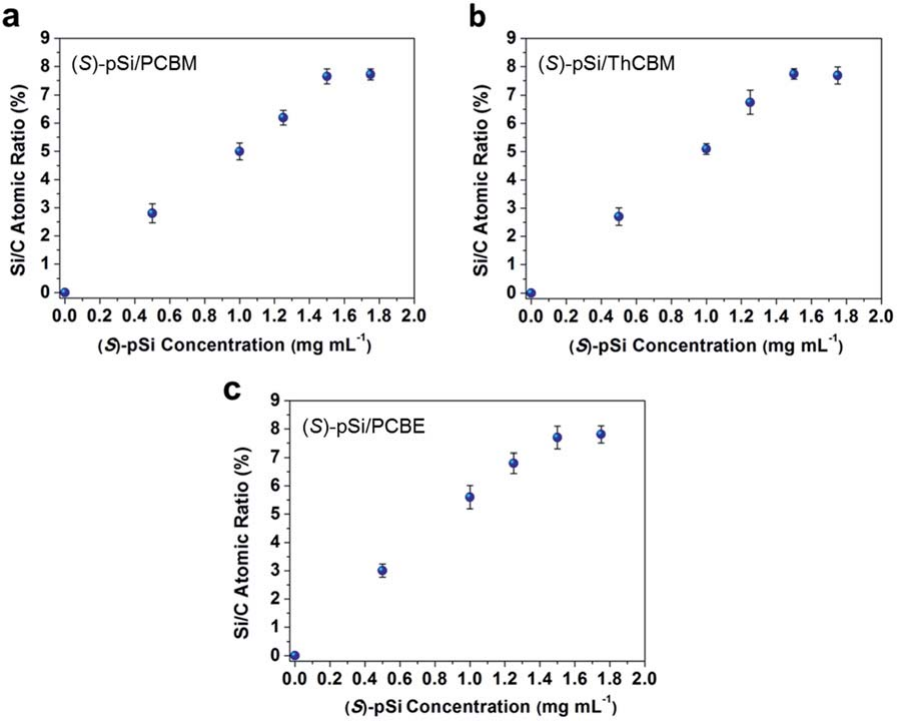
Si/C atomic ratios on the surfaces of (a) (*S*)-pSi/PCBM, (b) (*S*)-pSi/ThCBM, and (c) (*S*)-pSi/PCBE films plotted as a function of the (*S*)-pSi concentrations in the blend solutions. The concentrations of PCBM, ThCBM, and PCBE were fixed at 10 mg mL^−1^. All the films were spin-coated on silicon wafers, and then annealed at 150 °C for 30 min before XPS measurements.

The surface segregation behavior of (*S*)-pSi in the fullerene derivative films was systematically investigated by X-ray photoelectron spectroscopy (XPS) according to our previous method.^36,38,40,42^ The concentration of the fullerene derivative in the blend solutions was fixed as 10 mg mL^−1^ and the concentrations of the chiral surface modifiers were varied from 0 to 1.75 mg mL^−1^. The spin-coated films were thermally annealed under N_2_ at 150 °C for 30 min before XPS measurements. The Si/C atomic ratios on the film surface were calculated from the intensities of the Si 2p and C 1s peaks in the XPS spectra. Fig. 3 shows the Si/C ratios on the surfaces of the (*S*)-pSi/PCBM, (*S*)-pSi/ThCBM, and (*S*)-pSi/PCBE films as a function of the concentration of (*S*)-pSi in the solutions. The Si/C ratios depended linearly on the concentration of (*S*)-pSi in the lower concentration region up to 1.5 mg mL^−1^ and become constant at above that. This behavior is similar to previous studies on SSM formation^42^ and suggests that the surface energy of (*S*)-pSi is low enough for all the added (*S*)-pSi molecules to adsorb to the air/liquid interface during spin-coating and remain on the film surface. The Si/C ratio at the saturation point was close to that of pSi/PCBM (7.5%), at which the surface coverage with pSi is estimated to be higher than 90%.^42^ These results indicate that (*S*)-pSi was segregated to the surface of the films of PCBM, ThCBM, and PCBE to form the SSMs (Fig. 2). This was further confirmed by the results of XPS depth profiles and angle-resolved XPS (Fig. S1–S3†). The depth profiles showed that the high surface coverages with (*S*)-pSi SSMs can be maintained with a fixed surface modifier concentration (1.5 mg mL^−1^) in the solutions while the total film thicknesses can be independently controlled with the concentrations of the fullerene derivatives for the bulk of the films. The thicknesses of the oligosiloxane layers on the surface were estimated as 1.18–1.25 nm by the angle-resolved XPS analysis based on a bilayer model. The concentration of the chiral surface modifiers close to the saturation point (1.5 mg mL^−1^) was used in subsequent experiments to ensure the maximum SSM coverage.

The surface Si/C ratios of the films with an (*S*)-pSi concentration of 1.5 mg mL^−1^ after annealing at different temperatures (120–160 °C) were measured by XPS (Fig. S4†). The Si/C ratios showed very little change after annealing, regardless of the temperature, indicating that all the (*S*)-pSi molecules had already segregated to the film surface of the as-cast films and that thermal annealing did not substantially change the density or the molecular orientation of (*S*)-pSi at the surface.

### Crystallization induced by SSM

**Fig. 4:**
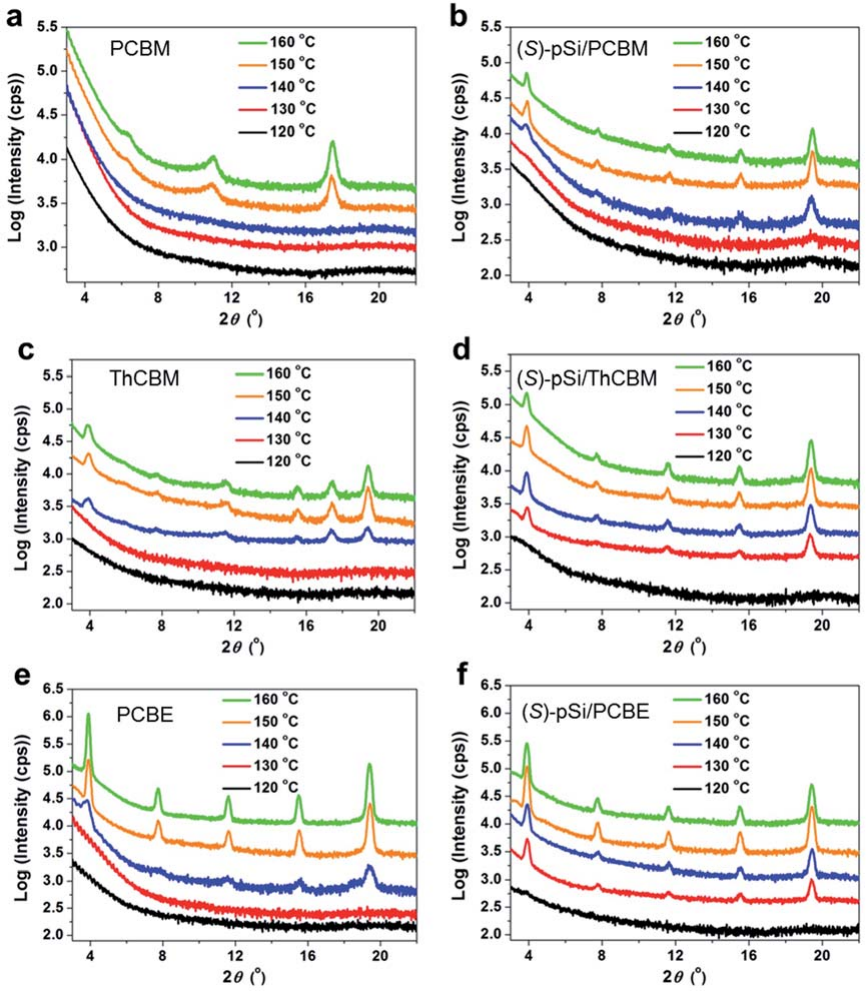
Out-of-plane XRD patterns of pure (a) PCBM, (c) ThCBM, and (e) PCBE films annealed at different temperatures. The films were prepared by spin-coating the fullerene derivative solutions (10 mg mL^−1^). Out-of-plane patterns of (b) (*S*)-pSi/PCBM, (d) (*S*)-pSi/ThCBM, and (f) (*S*)-pSi/PCBE annealed at different temperatures. The films were prepared by spin-coating the (*S*)-pSi (1.5 mg mL^−1^) and fullerene derivative (10 mg mL^−1^) blend solutions.

Fig. 4a shows the out-of-plane X-ray diffraction (XRD) patterns of the pure PCBM film after annealing at different temperatures. There were no clear peaks in the patterns for the as-cast PCBM films and the PCBM films annealed below 140 °C, indicating an amorphous structure. After annealing above 150 °C, the patterns showed peaks at 10.9° and 17.5°, which is consistent with the reported unsolved structure of PCBM films formed through spontaneous cold crystallization.^44,45^Fig. 4b shows XRD patterns of (*S*)-pSi/PCBM films after thermal annealing at different temperatures. The characteristic diffraction peaks at 3.9°, 7.8°, 11.7°, 15.6°, and 19.5° appeared after annealing above 140 °C, suggesting that the crystal phase formation was induced by the SSM, as reported for pSi/PCBM, and the peaks can be assigned to 002, 004, 006, 008 and 0010, respectively.^42^ The peak intensities of the surface-induced phase reached a maximum after annealing at 150 °C and increasing the annealing temperature to 160 °C did not change the intensities. 2D grazing-incidence wide-angle X-ray scattering (GIWAXS) measurements of the crystallized films showed similar diffraction patterns for the (*S*)-pSi/PCBM and pSi/PCBM films (Fig. S5†). These results indicate that the (*S*)-pSi SSM induced the PCBM crystallization in a similar way to the pSi SSM.^42^ The pure (*S*)-pSi film after annealing at 150 °C showed only halos in the GIWAXS pattern (Fig. S5a†), suggesting that it was amorphous.

The crystallization behavior of ThCBM and PCBE in the films was different from that of PCBM. The pure ThCBM films after thermal annealing showed the same diffraction peaks as the (*S*)-pSi/PCBM film at 3.9°, 7.8°, 11.7°, 15.6°, and 19.5° when the annealing temperature was above 140 °C (Fig. 4c). In addition, an extra peak appeared at 17.4°, which could not be assigned to the reported crystal structure of the SSM-induced phase. This extra peak may have the same origin for the peak of PCBM film after the cold crystallization (17.5°). This result indicates that pure ThCBM films underwent spontaneous cold crystallization with a packing motif similar to that of the SSM-induced phase of PCBM with possible mixed crystal phases. The (*S*)-pSi SSM at the film surface lowered the lowest crystallizing temperature of ThCBM from 140 to 130 °C (Fig. 4d). The diffraction patterns of the (*S*)-pSi/ThCBM films were similar to that of the pure ThCBM film, although the extra peak at 17.4° was not observed. In addition, the (*S*)-pSi/ThCBM films had higher intensities and sharper peaks compared with the pure ThCBM films, most notably at an annealing temperature of 150 °C. These results suggest that even though there was no drastic change in the crystal structure, as for (*S*)-pSi/PCBM, the (*S*)-pSi SSMs induced the crystallization of ThCBM into the pure phase at lower temperatures and increased the film crystallinity. The 2D GIWAXS measurements confirmed that the packing motifs of ThCBM in (*S*)-pSi/ThCBM and PCBM in (*S*)-pSi/PCBM were similar (Fig. S5c and f†).

The (*S*)-pSi/PCBE film showed crystallization behavior similar to that of the (*S*)-pSi/ThCBM film. The pure PCBE film started to crystallize after annealing at 140 °C, and a high diffraction peak intensity was observed after annealing above 150 °C, indicating high crystallinity (Fig. 4e). Fig. 4f shows the XRD patterns of (*S*)-pSi/PCBE films after annealing at different annealing temperatures. The (*S*)-pSi/PCBE film showed a lower crystallization transition temperature (130 °C) compared with that for the pure PCBE film (140 °C). In contrast to ThCBM, the diffraction peak intensities of the pure PCBE film were higher than those of (*S*)-pSi/PCBE, suggesting that the SSM did not improve the crystallinity of PCBE, even though it lowered the crystallization temperature. The 2D GIWAXS patterns of the PCBE and (*S*)-pSi/PCBE films were similar to those of the pSi/PCBM, (*S*)-pSi/PCBM, and (*S*)-pSi/ThCBM films (Fig. S5†), indicating that all these films had similar packing motifs.

**Fig. 5:**
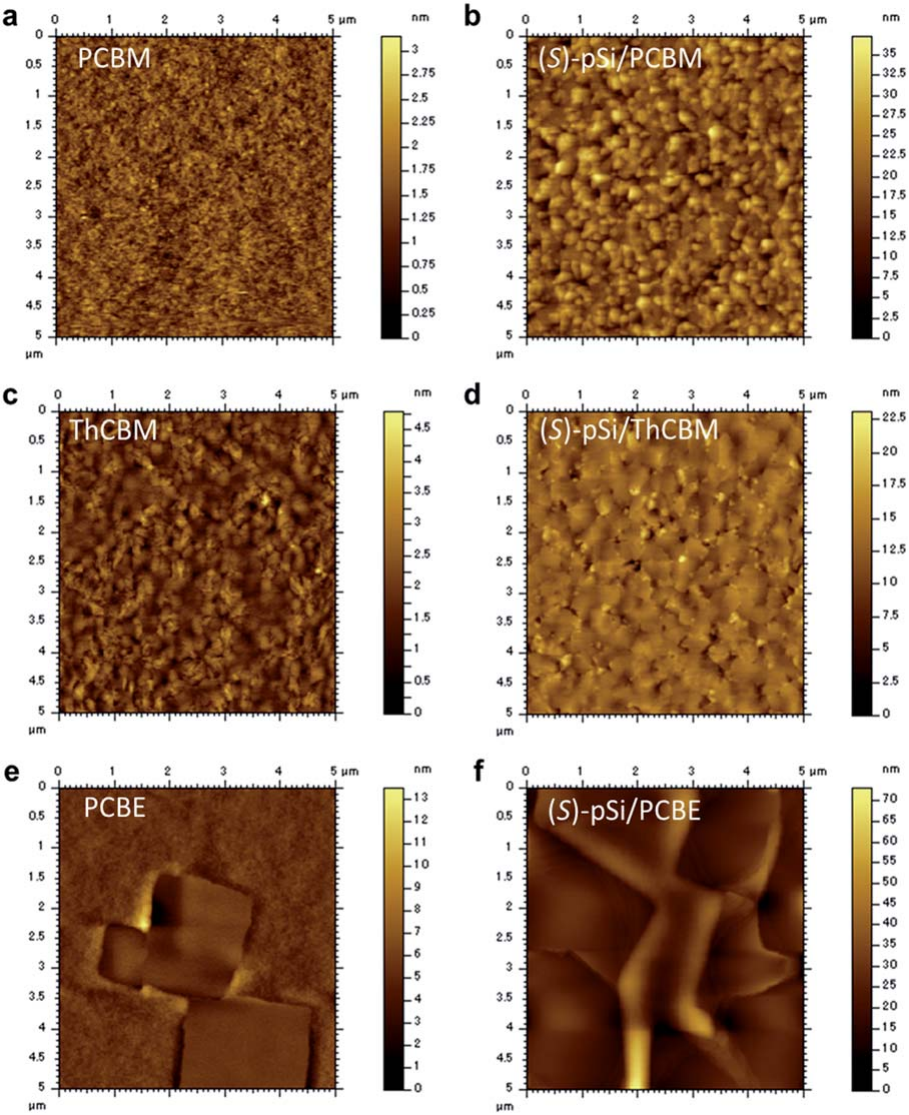
AFM height images of (a) PCBM, (b) (*S*)-pSi/PCBM, (c) ThCBM, (d) (*S*)-pSi/ThCBM, (e) PCBE, and (f) (*S*)-pSi/PCBE films after annealing at 150 °C for 30 min.

Atomic force microscopy (AFM) was performed to investigate the morphology of the crystallized fullerene films with and without the SSMs. The as-cast (*S*)-pSi/PCBM film was very flat with a root-mean-square roughness (*R*_q_) of 0.20 nm (Fig. S6†). After annealed at 150 °C for 30 min, although the pure PCBM film had a very flat surface with *R*_q_ of 0.31 nm (Fig. 5a), the (*S*)-pSi/PCBM film had larger grains with a larger *R*_q_ of 4.3 nm (Fig. 5b). This could reflect the differences in the crystal phases and the crystallinity, as revealed by XRD and GIWAXS. The pure ThCBM film had a grained surface structure (Fig. 5c) because the film was crystallized by thermal annealing. The (*S*)-pSi/ThCBM film had a similar surface structure, but with larger polygonal grains that were more distinct (Fig. 5d). The *R*_q_ values of ThCBM and (*S*)-pSi/ThCBM were 0.49 and 1.86 nm, respectively. The pure PCBE film showed large square grains on the surface (Fig. 5e) owing to the high crystallinity of the films and the tetragonal crystal structure. The (*S*)-pSi/PCBE film had similar square grains with a higher surface roughness (Fig. 5f). The *R*_q_ values of PCBE and (*S*)-pSi/PCBE were 1.1 and 4.1 nm, respectively.

### Induced chirality

**Fig. 6:**
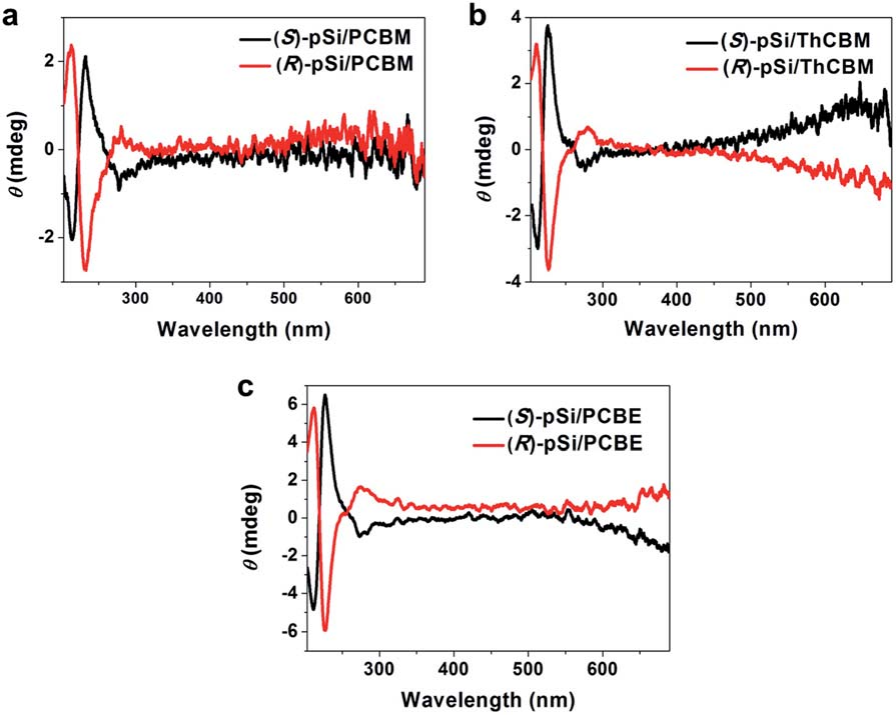
CD spectra of (a) (*S* or *R*)-pSi/PCBM, (b) (*S* or *R*)-pSi/ThCBM, and (c) (*S* or *R*)-pSi/PCBE films. The films were prepared by spin-coating the blend solutions of the chiral surface modifier (1.5 mg mL^−1^) and fullerene derivative (10 mg mL^−1^). After spin-coating, the films were annealed at 150 °C for 30 min.

Circular dichroism (CD) spectroscopy was used to investigate the induced chirality in the fullerene derivative films with (*S*)-pSi and (*R*)-pSi SSMs. The films for the CD measurements were prepared by spin coating on fused quartz substrates. After spin-coating, the films were annealed at 150 °C for 30 min to crystallize the fullerene derivatives. All the PCBM, ThCBM, and PCBE films with (*S*)-pSi and (*R*)-pSi SSMs showed clear cotton effects in the CD spectra (Fig. 6). The signs of the signals were inverted depending on the enantiomeric structure of the surface modifier. The signal positions in the CD spectra were correlated with the strong UV-vis absorption bands of PCBM, ThCBM, and PCBE at around 220 and 270 nm (Fig. S7†). To exclude the possible effects of optical anisotropy of the films on the CD signals, the samples were rotated to the incident light axis or flipped the faces in the CD measurements (Fig. S8†). The operations did not change the CD signal intensity, and linear dichroism (LD) signals remained silent regardless of the rotation angle. These results indicated that the CD activities are originated from the chiral structure in the film, not the optical anisotropy.^46–48^ Before thermal annealing, the (*S* or *R*)-pSi/PCBM, (*S* or *R*)-pSi/ThCBM, and (*S* or *R*)-pSi/PCBE films were amorphous and completely silent in the CD spectra (Fig. S9†). These results indicate that the chirality of the films appeared during crystallization under thermal annealing. In addition, chloroform solutions of (*S*)-pSi and (*R*)-pSi (Fig. S10a†) and the pure amorphous films of (*S*)-pSi and (*R*)-pSi after annealing at 150 °C for 30 min showed no signal in CD spectra (Fig. S10b†). These control experiments suggest that the CD signals observed in the films with the SSMs did not originate from the chiral surface modifiers themselves, but from the crystallized achiral fullerene derivatives (PCBM, ThCBM, and PCBE) inside the films. Notably, no CD signal was observed when a 1 : 1 mixture of (*S*)-pSi and (*R*)-pSi was used as surface modifier to crystallize fullerene film (Fig. S11†).

**Fig. 7:**
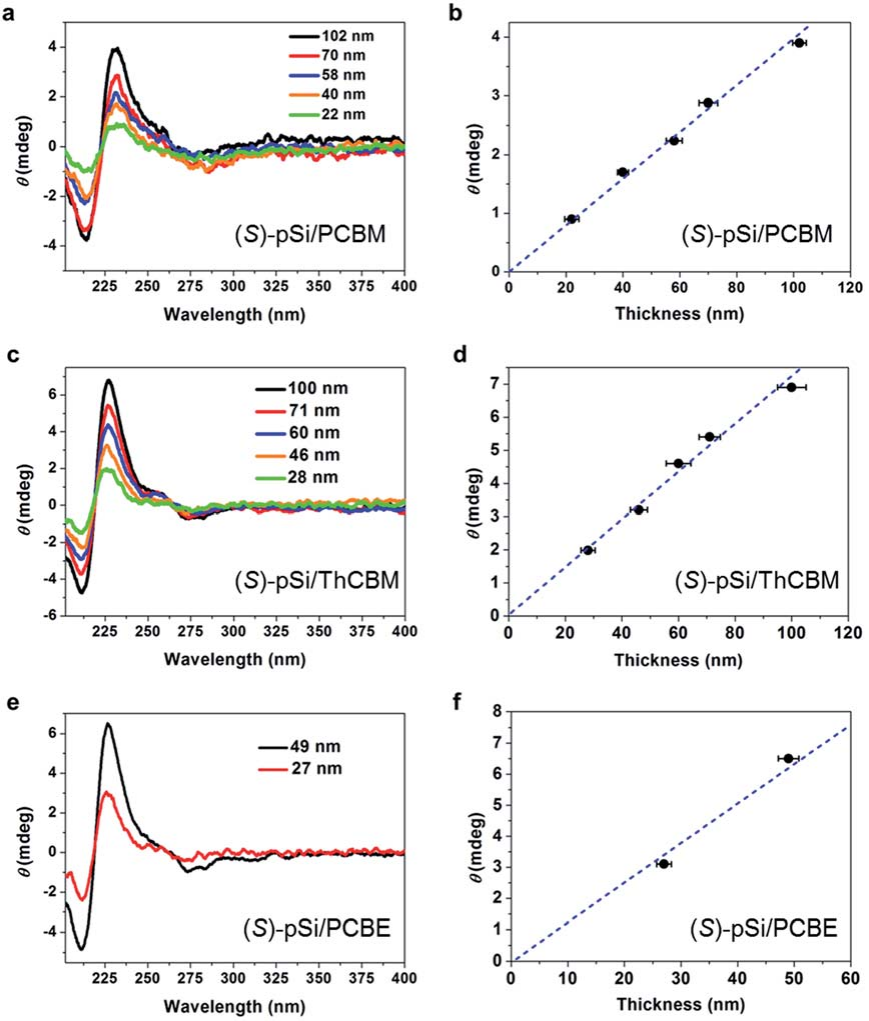
CD spectra of (a) (*S*)-pSi/PCBM, (c) (*S*)-pSi/ThCBM, and (e) (*S*)-pSi/PCBE films with different thicknesses. Ellipticity of the positive peak at 230 nm in the CD spectra of (b) (*S*)-pSi/PCBM, (d) (*S*)-pSi/ThCBM, and (f) (*S*)-pSi/PCBE films plotted as a function of the film thickness. Dashed lines are the linear regressions of the data.

To confirm the source of chirality in the fullerene films, the thickness dependence of the CD spectra was investigated. The (*S*)-pSi/PCBM films were prepared with a fixed (*S*)-pSi concentration of 1.5 mg mL^−1^ and PCBM concentrations of 5, 10, 12.5, 15, and 20 mg mL^−1^ to maintain the surface coverage with (*S*)-pSi SSMs while the total film thickness increased. The films were spin-coated from the blend solutions and annealed at 150 °C for 30 min, and the thicknesses of the films were measured by surface profilometry. Fig. 7a shows the CD spectra of (*S*)-pSi/PCBM films with different film thicknesses and Fig. 7b shows the ellipticity of the peak at 230 nm plotted as a function of the thickness. The ellipticity increased almost linearly as the thickness of the films increased from 22 to 102 nm. The absorbance of the films had a linear relationship with the thickness of the films in this range, indicating that the films had a uniform density and structure in the vertical direction (Fig. S12b†). These results confirmed that the CD signals originated from the crystallized PCBM inside the films, not from the surface or the interfaces between the samples and the substrates. In addition, the good linearity suggests that the enantiomorphic crystals grew from the top to the bottom of the films, with a thickness of at least 100 nm.

The thickness dependence for (*S*)-pSi/ThCBM was also investigated in a similar way. Fig. 7c shows the CD spectra of these films, and the ellipticity of the peaks at 230 nm in the CD spectra were plotted as a function of film thickness (Fig. 7d). The CD intensity increased linearly as the film thickness increased from 28 to 100 nm. For (*S*)-pSi/PCBE films, due to the low solubility of PCBE, only 5 and 10 mg mL^−1^ PCBE with 1.5 mg mL^−1^ (*S*)-pSi blend solutions were used. Nevertheless, a clear increase in CD intensity was also observed for (*S*)-pSi/PCBE films when the film thickness increased from 27 to 49 nm (Fig. 7e and f).

The dependence of the ellipticity on the thickness can give information on the factors that affect the chiral induction from the SSMs. The slopes of the linear fitting for the *θ*-thickness relationship were 0.038, 0.071, and 0.131 mdeg nm^−1^ for (*S*)-pSi/PCBM, (*S*)-pSi/ThCBM, and (*S*)-pSi/PCBE, respectively. This difference may be related to the crystallinity of the fullerene derivatives inside the films; the order of the crystallinity is PCBE > ThCBM > PCBM, as determined from the intensity of the XRD patterns in Fig. 4. The fraction of the crystallized domains or the disorder of the crystal structures inside the films could affect the intensity of the induced CD signals.

To further confirm the origin of the crystallization and the chirality, the surface layer of the as-cast (*S*)-pSi/PCBM film was removed by reactive-ion etching with O_2_ plasma and the resulted film was subsequently annealed at 150 °C for 30 min (Fig. S13†). XRD results showed that the films did not crystalize into the SSM-induced crystal phase after annealing, but into the ordinary crystal phase observed for the pure PCBM films. The films showed no CD signal. This control experiment provides the strong evidence that the crystallization and the chirality of the fullerene film was induced from the surface monolayer of (*S*)-pSi.

Our previous structural analysis on pSi/PCBM films based on GIWAXS patterns gave the crystal structure with the space group of *I*4̄*c*2 that is non-centrosymmetric but not chiral. Since the chiral (*S*)-pSi/PCBM crystal structure must belong to the chiral space group, but gives the identical GIWAXS pattern as pSi/PCBM films, there are two possible situations: (1) the crystallization induced by (*S*)-pSi SSM lowers the crystal symmetry from *I*4̄*c*2 into *I*4 or *I*422, or (2) pSi/PCBM and (*S*)-pSi/PCBM films have the same crystal structure with the space group of *I*4 or *I*422. In either case, *I*4 or *I*422 structure could show the additional diffraction peaks on the GIWAXS patterns resulting from disappearance of the *c*-glide symmetry of *I*4̄*c*2 space group. Unfortunately, however, the information from GIWAXS patterns of the 2-D random films is limited and detailed analysis on the patterns did not show any evidence of the lowered symmetry in either (*S*)-pSi/PCBM or pSi/PCBM films. This is possibly due to a pseudo *c*-glide symmetry between crystallographically independent PCBM molecules and/or the lack of the electron density contributing to the corresponding reflections. In the case of (2), the pSi/PCBM films have chiral crystal domains but they are 1 : 1 mixture of the two enantiomorphic structures. In this case, these domains may be observable by advanced imaging methods such as CD imaging. At this stage, we cannot conclude which situation is real, and further investigation on the actual crystal structure of the chiral (*S*)-pSi/PCBM film is necessary.

## Conclusions

We demonstrated that chiral SSMs of (*S*)-pSi and (*R*)-pSi induced the formation of enantiomorphic crystal structures of achiral fullerene derivatives in thin films. This is the first report of the surface-induced chirality of organic semiconductors in films during crystallization. This study provides a strategy to prepare chiral crystalline organic films and offers a better understanding of chiral induction. In addition, the resulting thin-film chiral materials may have interesting applications in nonlinear optics and spintronic devices.

## Experimental methods

### Materials

PCBM was purchased from Solenne BV (Netherlands). ThCBM and PCBE were purchased from ATR Company (Japan). The other chemicals and solvents were purchased from FUJIFILM Wako Pure Chemical (Japan), Sigma-Aldrich (USA), or TCI Chemicals Co. (Japan). All reagents were used as received, unless otherwise indicated. The synthesis and characterizations of the surface modifiers (*S*)-pSi and (*R*)-pSi are described in the ESI.† All moisture- or air-sensitive reactions were carried out under a nitrogen atmosphere by standard Schlenk techniques. Column chromatography was conducted using silica gel with a particle diameter of 20–40 μm.

### Sample preparation

Silicon wafers and quartz glass substrates were cleaned by sequential ultrasonication in detergent solution, water, acetone, and 2-propanol. The substrates were dried, and then subjected to UV-O_3_ treatment. The spin-coating solution was prepared by dissolving the fullerene derivative (5–20 mg) and surface modifiers (0–1.75 mg) in chloroform (1 mL). The solution was spin-coated on the substrates at 2500 rpm for 30 s under N_2_. The films were thermally annealed on a hotplate under N_2_ for 30 min.

### Characterization

^1^H and ^13^C NMR spectra were recorded on a 300 MHz spectrometer (JNM-AL300, JEOL, Japan). Data are reported as chemical shift in ppm (*δ*), multiplicity, coupling constant (Hz), integration, and assignments. High-resolution mass spectrometry was performed on a mass spectrometer (JMS-T100GCV, JEOL). XPS was performed with an X-ray spectrophotometer (PHI 5000 Versa Probe II, ULVAC-PHI, Japan). Monochromated Al Kα (1486.6 eV) radiation was used in all XPS measurements. The C 1s (285 eV), Si 2p (102 eV), and O 1s (532 eV) peaks were used in the characterizations. To obtain the XPS depth profile, each sample was etched with Ar^+^ ion at an acceleration voltage of 500 V with an etching rate of approximately 0.25 nm s^−1^. The CD and UV-vis absorption spectra of films on quartz glass substrates were recorded on a spectropolarimeter (J-820, JASCO, Japan) and spectrophotometer (V-670, JASCO), respectively. The film thickness was measured with a surface profilometer (Dektak 6 M, ULVAC-PHI). XRD measurements were performed on an X-ray diffractometer (Smartlab, Rigaku, Japan) with Cu Kα radiation (*λ* = 0.154 nm). GIWAXS measurements were conducted at beamline BL46XU of SPring-8, Japan. The irradiation wavelength for GIWAXS was *λ* = 0.10002 nm (energy: 12.398 keV) and the incident angle was fixed at 0.12°. AFM images were obtained with a scanning probe microscope (5400, Agilent Technologies, USA) in tapping mode.

## Conflicts of interest

The authors declare no conflict of interest.

## Supplementary Material

SC-011-D0SC01163K-s001
