# Supplementary material for: Surface-induced enantiomorphic crystallization of achiral fullerene derivatives in thin films
Source: Chem Sci. 2020 Apr 17;11(18):4702–8. doi: 10.1039/d0sc01163k (PMC8159225; doi:10.1039/d0sc01163k)
Supplement: SC-011-D0SC01163K-s001 [file SC-011-D0SC01163K-s001.pdf]

Electronic Supplementary Material (ESI) for Chemical Science.
This journal is © The Royal Society of Chemistry 2020

Supporting Information for

Surface-induced Enantiomorphic Crystallization of Achiral Fullerene Derivatives in Thin Films

Chao Wang[1], Hua Hao[1], Daisuke Hashizume[1] and Keisuke Tajima[1]*

[1]RIKEN Center for Emergent Matter Science (CEMS), 2-1 Hirosawa, Wako, Saitama 351-0198, Japan. E-mail: keisuke.tajima@riken.jp

Synthesis

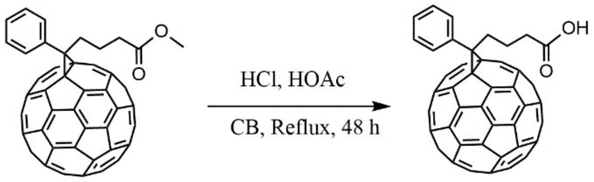

PCBM **PCBA**

PCBA. Acetic acid (50 mL) and hydrochloric acid (12 M, 20 mL) were added to a solution of PCBM (500 mg, 0.549 mmol) in chlorobenzene (100 mL). The reaction mixture was refluxed for 48 h and cooled to room temperature. The solvent was removed under reduced pressure and the residue was purified by precipitation from methanol to obtain PCBA as a brown solid (443 mg, 90%). ^{1}H NMR ($CS_2/CDCl_3$, 300 MHz): δ 7.93 (d, J = 7.6 Hz, 2H, Ar-H), 7.47-7.61 (m, 3H, Ar-H), 2.91-2.96 (m, 2H, -CH_2-CO-), 2.58 (t, J = 7.3 Hz, 2H, C-CH_2-C), 2.18-2.26 (m, 2H, C-CH_2-C).

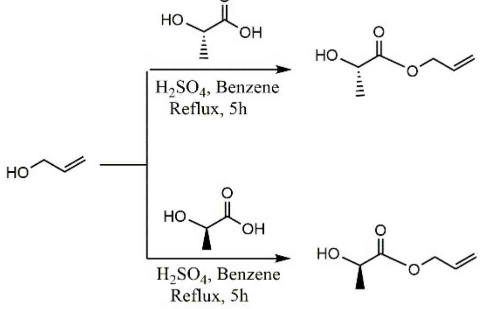

Allyl L-(−)-lactate. (*S*)-Lactic acid (85%, 4.0 g, 0.044 mol), benzene (15 mL), and concentrated sulfuric acid (0.05 mL) were placed in a 50 mL round-bottomed flask equipped with a Dean-Stark trap and a reflux condenser. The mixture was heated to reflux, and the water was removed by the Dean-Stark system. Allyl alcohol (10.2 g, 0.176 mol) was rapidly added, and refluxing and removal of water were continued for 5 h. The mixture was neutralized with anhydrous sodium acetate, and the product was purified by distillation under reduced pressure (4.3 g, 75%). ^{1}H NMR ($CDCl_3$, 300 MHz): δ 5.91 (ddt, J = 17.2, 10.3, 5.6 Hz, 1H, C-CH=C), 5.35 (dq, J = 17.2, 1.0 Hz, 1H, -C=CH), 5.27 (dq, J =10.3, 1.1 Hz, 1H, -C=CH), 4.66 (dt, J = 5.7, 1.1 Hz, 2H, -O-CH_2-C), 4.30 (q, J = 6.9 Hz, 1H, HO-CH-CO-), 2.92 (m, 1H, OH), 1.43 (d, J = 6.9 Hz, 3H, CH_3). ^{13}C NMR ($CDCl_3$, 300 MHz): δ 175.6, 131.6, 119.0, 66.8, 66.1, 20.3.

Allyl D-(+)-lactate. Allyl D-(+)-lactate was prepared from (*R*)-lactic acid by following the same procedure as for allyl L-(−)-lactate in 71% yield. The NMR data for allyl D-(+)-lactate are the same as for allyl L-(−)-lactate.

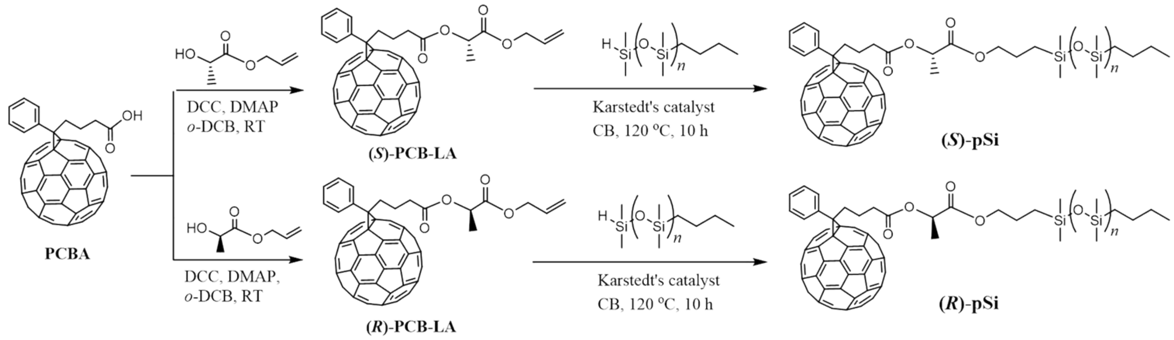

(*S*)-PCB-LA. *N*,*N'*-Dicyclohexylcarbodiimide (91.6 mg, 0.444 mmol) and 4-dimethylaminopyridine (54.2 mg, 0.444 mmol) were added to a solution of PCBA (200 mg, 0.223 mmol) and allyl L-(−)-lactate (31.2 mg, 0.24 mmol) in *ortho*-dichlorobenzene (40 mL) quickly at

0 °C in an ice/water bath. The mixture was stirred under N_2 at 0 °C for 1 h, and then warmed to room temperature and stirred overnight. After removal of the solvent under reduced pressure, the residue was purified by silica gel chromatography using toluene/hexane (2:1, v/v) followed by pure toluene as the eluent to give (*S*)-PCB-LA as a brown solid (138 mg, 62%). ^{1}H NMR ($CDCl_3$, 300 MHz): δ 7.94 (d, J = 6.9 Hz, 2H, Ar-H), 7.47-7.57 (m, 3H, Ar-H), 5.92 (ddt, J = 17.2, 10.4, 5.6 Hz, 1H, C-CH=C), 5.36 (dq, J = 17.2, 1.4 Hz, 1H, -C=CH), 5.27 (dq, J = 10.4, 1.1 Hz, 1H, -C=CH), 5.12 (q, J = 6.9 Hz, 1H, -O-CH-CO-), 4.65 (dt, J = 5.7, 1.1 Hz, 2H, -O-CH_2-C), 2.91-2.96 (m, 2H, -CH_2-CO-), 2.61 (t, J = 7.4 Hz, 2H, C-CH_2-C), 2.16-2.26 (m, 2H, C-CH_2-C), 1.50 (d, J = 7.1 Hz, 3H, CH_3). ^{13}C NMR ($CDCl_3$, 300 MHz): δ 172.58, 170.63, 149.00, 147.93, 146.03, 145.35, 145.31, 145.25, 145.20, 144.95, 144.83, 144.66, 144.59, 144.16, 143.92, 143.28, 143.14, 143.09, 142.40, 142.33, 142.29, 141.14, 140.90, 138.29, 137.72, 136.87, 132.26, 131.65, 128.56, 128.38, 118.87, 79.91, 68.67, 65.88, 51.89, 33.71, 33.60, 22.11, 16.89. HRMS (ESI) *m/z* $[M + Na]^+$ calcd. for $C_{77}H_{20}O_4Na$: 1031.1259; found: 1031.1256.

(*R*)-PCB-LA. **(*R*)-PCB-LA** was prepared from allyl D-(+)-lactate by following the same procedure as for **(*S*)-PCB-LA** in 65% yield. The NMR data for (*R*)-PCB-LA are the same as for **(*S*)-PCB-LA**. HRMS (ESI) *m/z* calcd. for $C_{77}H_{20}O_4Na$ ($[M + Na]^+$): 1031.1259; found: 1031.1257.

(*S*)-pSi. Platinum(0)-1,3-divinyl-1,1,3,3-tetramethyldisiloxane complex solution (Karstedt's catalyst, 2% Pt in xylene, 100 μL) was added to a solution of **(*S*)-PCB-LA** (120 mg, 0.12 mmol) and *n*-butyl/hydride-terminated polydimethylsiloxane (108 mg; M_w: 800–900, GELEST, Inc., USA) in anhydrous chlorobenzene (5 mL). The reaction mixture was stirred under N_2 at 120 °C

for 10 h. After removal of the solvent under reduced pressure, the residue was purified by silica gel chromatography using toluene/hexane (1:1, v/v) as the eluent to give (*S*)-pSi as a brown solid (37 mg, 17%). ^{1}H NMR ($CDCl_3$, 300 MHz): δ 7.94 (d, J = 6.9 Hz, 2H, Ar-H), 7.44-7.56 (m, 3H, Ar-H), 5.09 (q, 1H, J = 7.1 Hz, -O-CH-CO-), 4.11 (m, 2H, -O-CH_2-C), 2.91-2.96 (m, 2H, -CH_2-CO-), 2.60 (t, J = 7.4 Hz, 2H, C-CH_2-C), 2.18-2.26 (m, 2H, C-CH_2-C), 1.61-1.71 (m, 2H, -O-C-CH_2-C-Si-), 1.48 (d, 3H, J = 6.9 Hz, -O-C-CH_3), 1.27-1.33(m, 4H, -Si-C-CH_2CH_2-C), 0.88 (t, 3H, J = 6.9 Hz, -C-C-CH_3), 0.50-0.56 (m, 4H, -CH_2-Si), 0.04-0.1 (m, 62H, Si-CH_3). ^{13}C NMR ($CDCl_3$, 300 MHz): 172.56, 170.97, 149.02, 147.95, 146.04, 145.36, 145.32, 145.26, 145.20, 144.96, 144.84, 144.67, 144.60, 144.17, 143.93, 143.28, 143.19, 143.15, 143.10, 142.41, 142.34, 142.30, 141.15, 140.90, 138.30, 137.73, 136.88, 132.27, 128.56, 128.38, 79.93, 68.68, 67.85, 51.91, 33.72, 33.61, 26.33, 25.41, 22.48, 22.10, 17.91, 16.95, 13.86, 13.76, 1.10, 0.99, 0.04. HRMS (ESI) *m/z* calcd. for $C_{97}H_{78}O_{11}Si_8Na$ (n = 7) ([M + Na]$^+$): 1666.3619; found: 1666.3597.

(*R*)-pSi. **(*R*)-pSi** was prepared from **(*R*)-PCB-LA** by following the same procedure as for **(*S*)-pSi** in 14% yield. The NMR data for **(*R*)-PCB-LA** are the same as for **(*S*)-PCB-LA**. HRMS (ESI) *m/z* calcd. for $C_{97}H_{78}O_{11}Si_8Na$ (n = 7) ([M + Na]$^+$): 1666.3619; found: 1666.3634.

Supplementary Figures

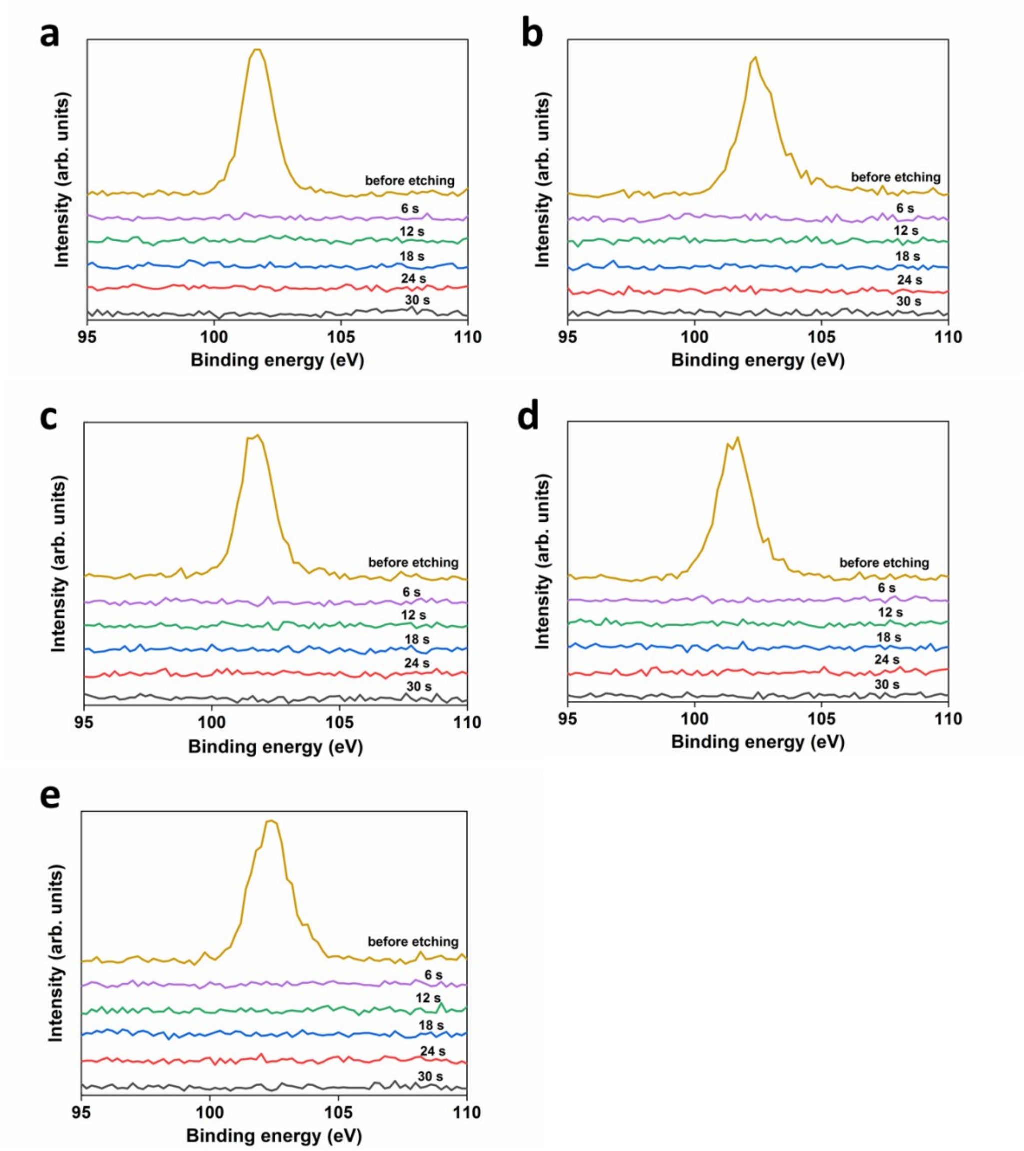

Figure S1. XPS depth profiles of Si 2p peak. XPS depth profiles of the Si 2p peak for (a) (*S*)-pSi/PCBM (45 nm), (b) (*S*)-pSi/PCBM (100 nm), (c) (*S*)-pSi/ThCBM (48 nm), (d) (*S*)-pSi/ThCBM (97 nm), and (e) (*S*)-pSi/PCBE (47 nm) films. The films of (a, c, e) were prepared by spin-coating the blend solutions of (*S*)-pSi (1.5 mg mL^{-1}) and a fullerene derivative (10 mg mL^{-1}). The films of (b, d) were prepared by spin-coating the blend solutions of (*S*)-pSi (1.5 mg mL^{-1}) and a fullerene derivative (20 mg mL^{-1}). All the films were thermally annealed at 150 °C for 30 min. Each sample was etched with an Ar^+ ion beam for several cycles (etching rate: 0.25 nm/s). The results showed that the Si 2p peak of all the films disappeared after the surface etching for 6 s, indicating that the oligosiloxane chains of (*S*)-pSi segregated to the surface as monolayers.

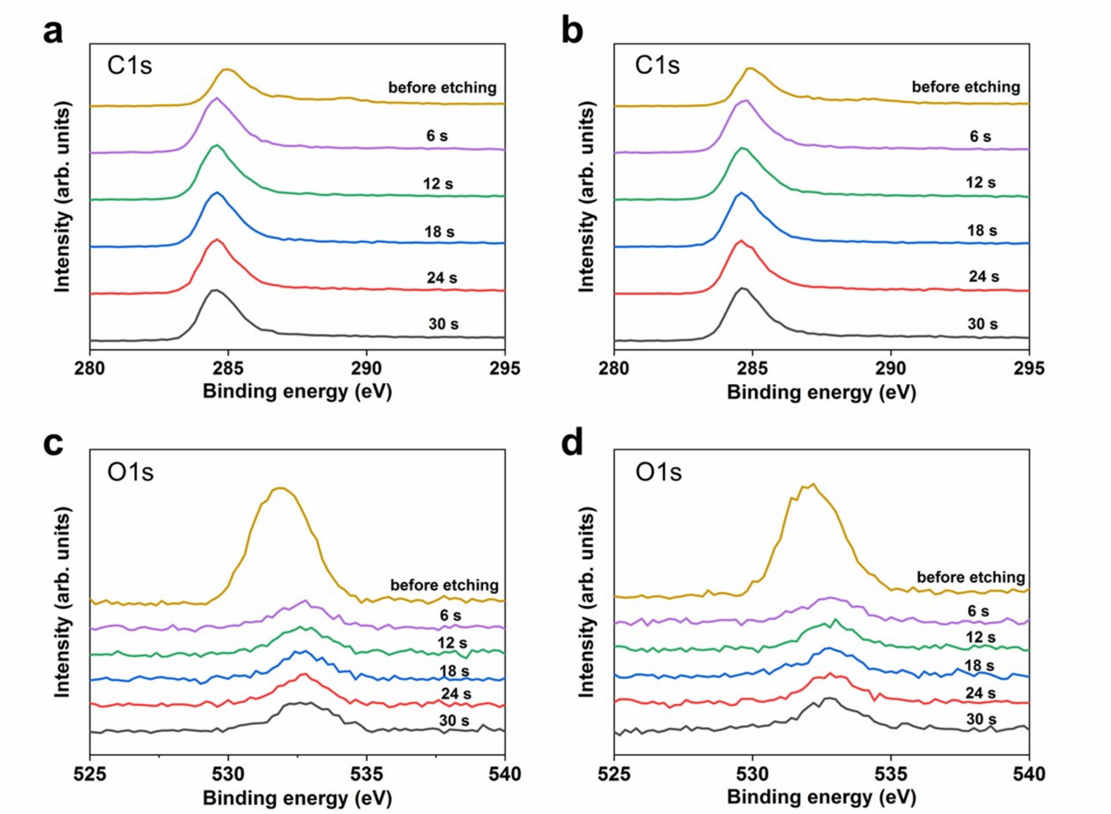

Figure S2. XPS depth profiles of C 1s and O 1s peaks. XPS depth profiles of the C 1s peak for (a) (*S*)-pSi/PCBM (45 nm) and (b) (*S*)-pSi/PCBM (100 nm) films (these films are the same as those for Figure S1a and b). Before etching, the main C 1s peak positioned at 285 eV can be deconvoluted to the peaks with the maxima at 284.4, 284.8 and 285.6 eV which can be assigned to C-Si, C=C and C-C, respectively. The peak top is shifted to 284.8 eV after etching for 6 s and remained constant after the longer etching time. This position is consistent with the C 1s peak of C=C in PCBM previously reported.[1, 2] XPS depth profiles of the O 1s peak of (c) (*S*)-pSi/PCBM (45 nm) and (d) (*S*)-pSi/PCBM (100 nm) films. Before etching, O 1s peak top was positioned at 532 eV, which can be mainly assigned to O-Si in oligo(dimethylsiloxane) (532.0 eV for poly(dimethylsiloxane)). The peak top is shifted to 532.7 eV after etching for 6 s and remained constant after the longer etching time. This position is consistent with the O 1s peaks of C=O and C-O-C in PCBM previously reported. [1, 2] The results showed that the SSM layer was removed after the etching for 6 s and only PCBM was observed in the bulk of the films.

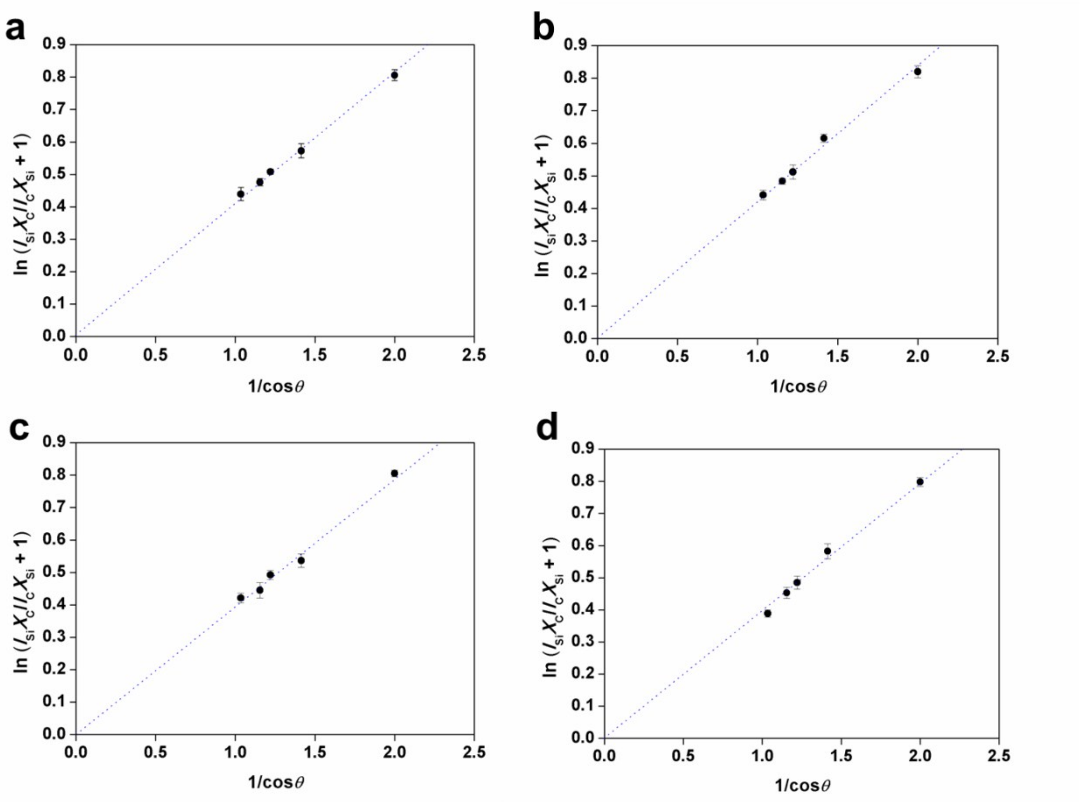

Figure S3. **Angle-resolved XPS**. Plot of $1/\cos\theta$ vs ln ($I_{Si}X_C$/ I_CX_{Si} + 1) from the angle-resolved XPS spectra in the (a) (*S*)-pSi/PCBM (45 nm), (b) (*S*)-pSi/PCBM (100 nm), (c) (*S*)-pSi/ThCBM (48 nm), and (d) (*S*)-pSi/ThCBM films (97 nm). The films of (a, c) were prepared by spin-coating the blend solutions of (*S*)-pSi (1.5 mg mL^{-1}) and a fullerene derivative (10 mg mL^{-1}). The films of (b, d) were prepared by spin-coating the blend solutions of (*S*)-pSi (1.5 mg mL^{-1}) and a fullerene derivative (20 mg mL^{-1}). The films were thermally annealed at 150 °C for 30 min prior to the measurements. The plots of ln ($I_{Si}X_C/I_CX_{Si}$ + 1) as a function of $1/\cos\theta$ were fitted well by straight lines using equation $\ln (I_{Si}X_C/I_CX_{Si} + 1) = d/\lambda\cos\theta$, indicating that the continuous surface segregated monolayers of (*S*)-pSi formed on the film surface of the fullerene derivatives. The equation $\ln (I_{Si}X_C/I_CX_{Si} + 1) = d/\lambda\cos\theta$ correlates with a uniform bilayer model [3] that consists of the oligosiloxane and the fullerene layers. In this equation, I_{Si} and I_C are the intensities of Si 2p and C 1s peaks, λ is the attenuation length of photoelectrons, θ is the take-off angle of the measurements, and X_{Si} and X_C are the local concentrations of silicon and carbon atoms, respectively. According to our previous report [4], the attenuation lengths of photoelectrons from C 1s and Si 2p are set to 3.0 nm, X_{Si} was calculated from the number of silicon atoms and the length of oligosiloxane chain, which was obtained from optimized structure in the DFT calculation, as 7.91 in both (S)-pSi/PCBM and (S)-pSi/ThCBM films. X_C was calculated from fullerene derivative carbon number/fullerene derivative length, as 57.1 for PCBM and 55.6 for ThCBM. The thickness of the oligosiloxane layer (*d*) can be calculated from the slope of these lines. The results were 1.24 nm for (a), 1.25 nm for (b), 1.21 nm for (c), and 1.18 nm for (d).

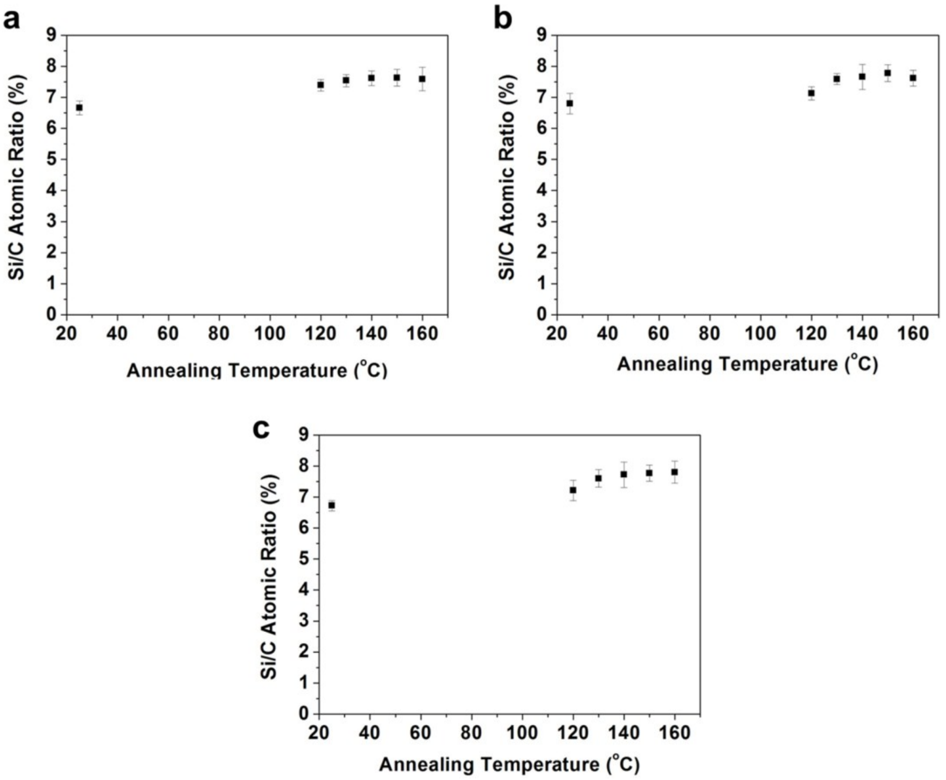

Figure S4. Annealing temperature dependence of Si/C atomic ratio. Si/C atomic ratio on the surfaces of the (a) (*S*)-pSi/PCBM, (b) (*S*)-pSi/ThCBM, and (c) (*S*)-pSi/PCBE films plotted as a function of the thermal annealing temperature. The films were prepared by spin-coating the blend solutions of the fullerene derivative (10 mg mL^{-1}) and surface modifier (*S*)-pSi (1.5 mg mL^{-1}) on silicon wafers.

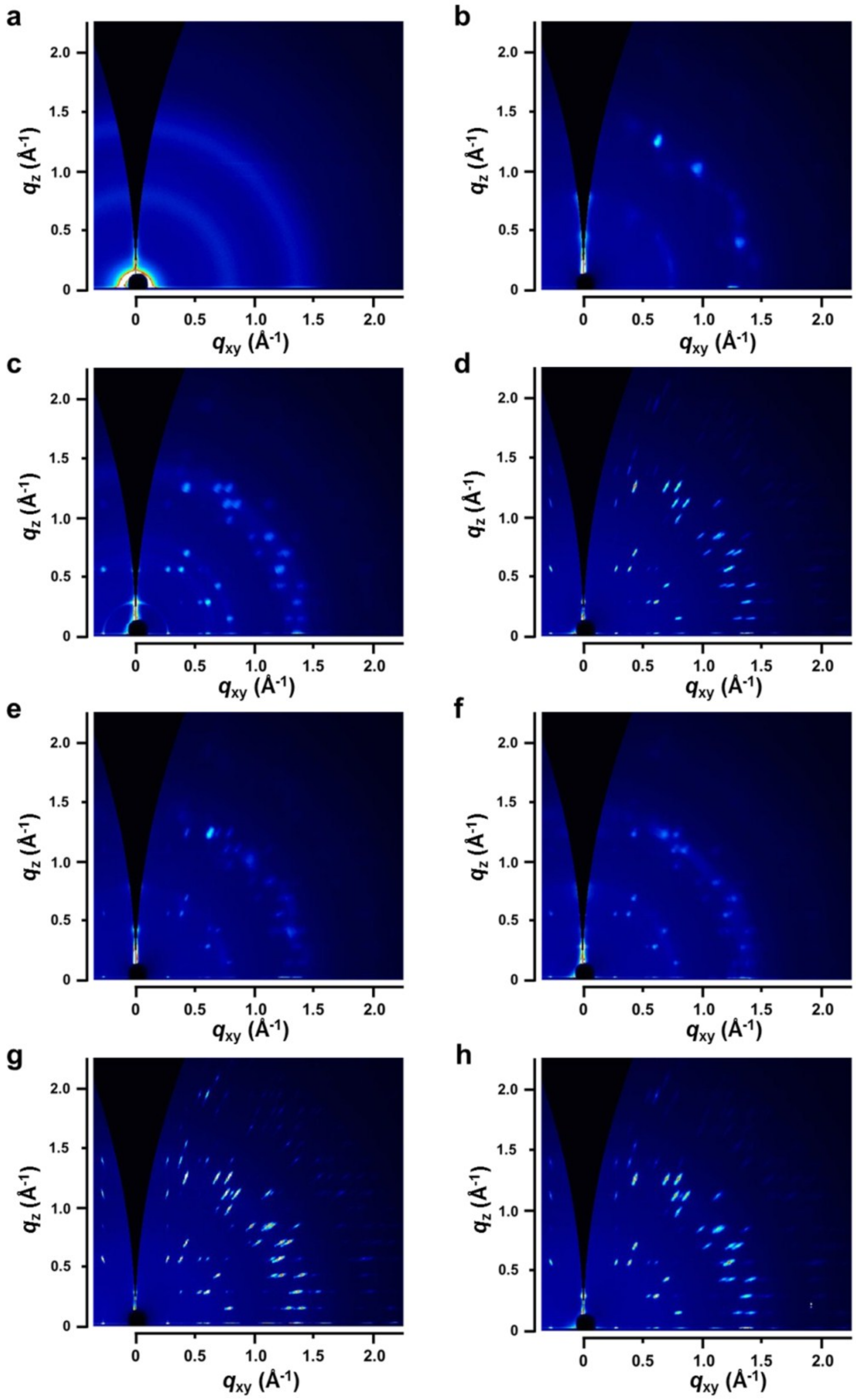

Figure S5. **GIWAXS patterns.** GIWAXS patterns of (a) pure (*S*)-pSi, (b) pure PCBM, (c) (*S*)-pSi/PCBM, (d) pSi/PCBM, (e) pure ThCBM, (f) (*S*)-pSi/ThCBM, (g) pure PCBE, and (h) (*S*)-pSi/PCBE films on silicon wafers. All the films were annealed at 150 °C for 30 min after spin-coating.

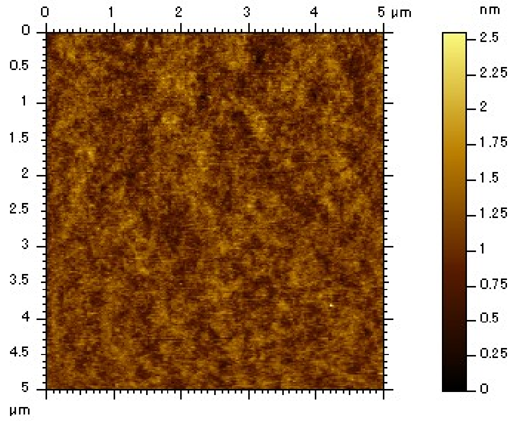

Figure S6. AFM height image of as-cast (*S*)-pSi/PCBM film. The film has a very flat surface with R_q of 0.2 nm.

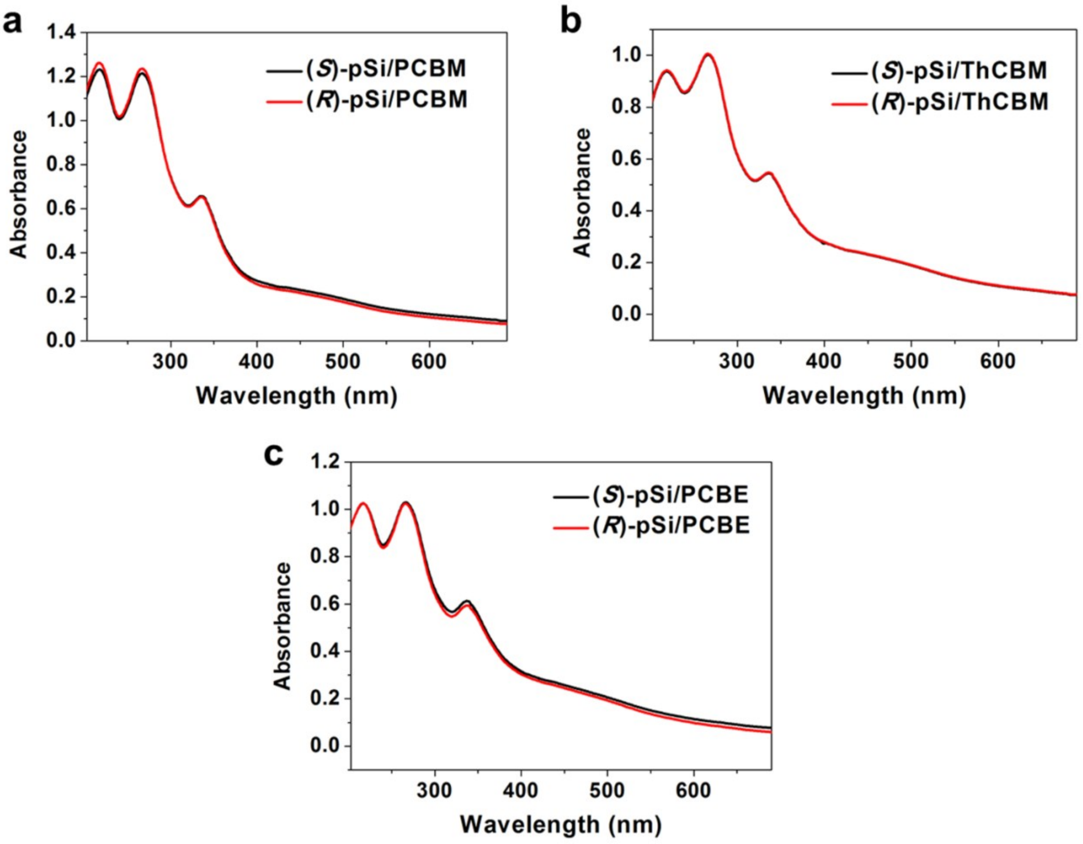

Figure S7. UV-Vis spectra of fullerene derivative films with SSMs. UV-Vis spectra for (a) (*S*)-pSi/PCBM and (*R*)-pSi/PCBM, (b) (*S*)-pSi/ThCBM and (*R*)-pSi/ThCBM, and (c) (*S*)-pSi/PCBE and (*R*)-pSi/PCBE. All the films were prepared by spin-coating the blend solutions of the chiral surface modifier (1.5 mg mL^{-1}) and a fullerene derivative (10 mg mL^{-1}). After spin-coating, all the films were annealed at 150 °C for 30 min.

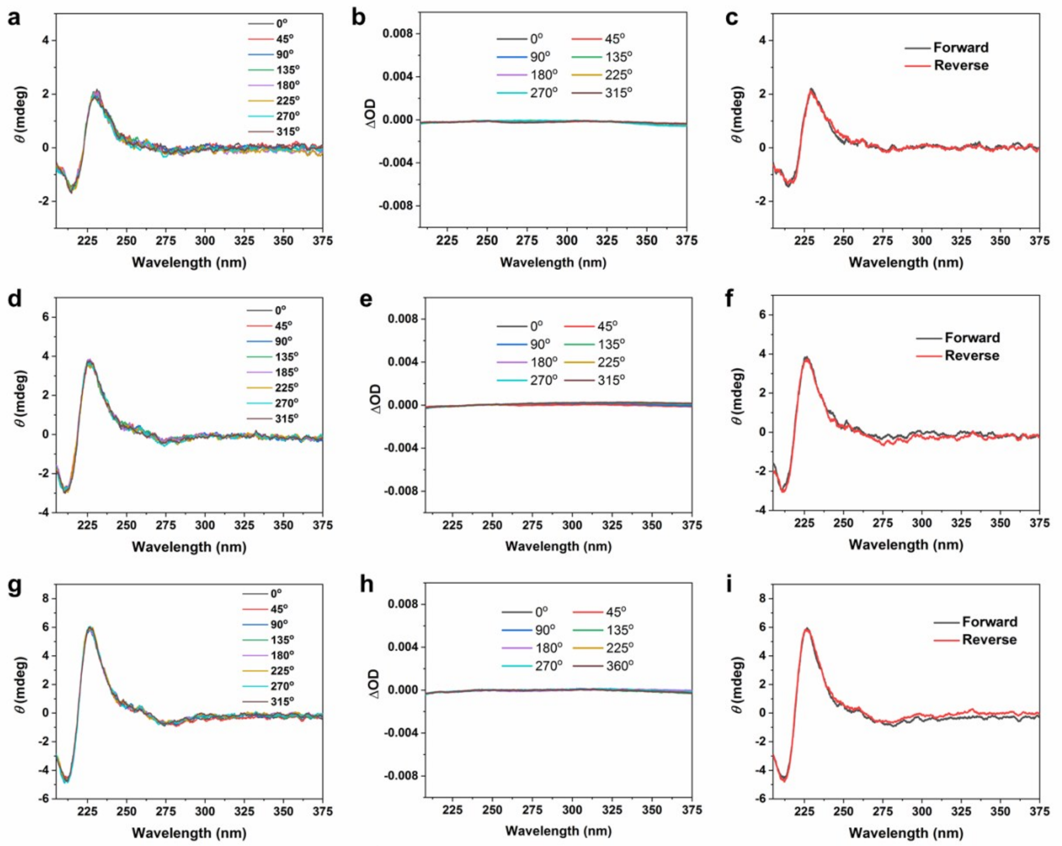

Figure S8. CD spectra of (a) (*S*)-pSi/PCBM, (d) (*S*)-pSi/ThCBM, and (g) (*S*)-pSi/PCBE films at various rotation angles. LD spectra of (b) (S)-pSi/PCBM, (e) (S)-pSi/ThCBM, and (h) (S)-pSi/PCBE films at various rotation angles. Forward and reverse CD spectra of (c) (*S*)-pSi/PCBM, (f) (*S*)-pSi/ThCBM, and (i) (*S*)-pSi/PCBE films. All the films were prepared by spin-coating the blend solutions of (*S*)-pSi (1.5 mg mL^{-1}) and a fullerene derivative (10 mg mL^{-1}). After spin-coating, all the films were annealed at 150 °C for 30 min.

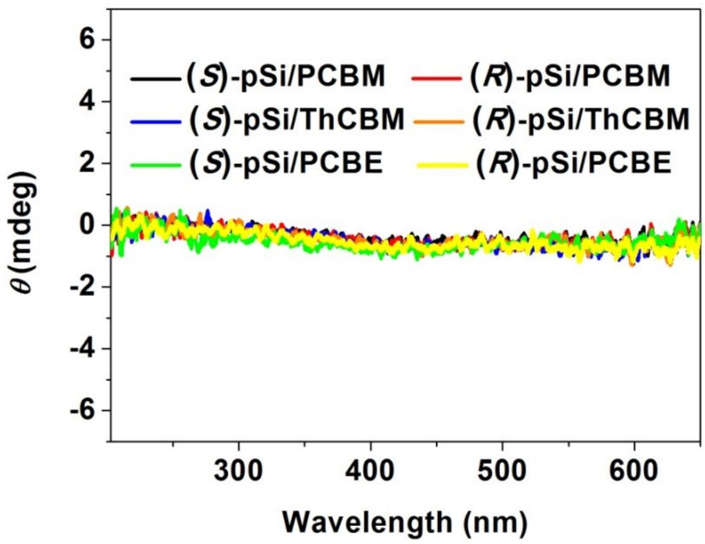

Figure S9. CD spectra before thermal annealing. CD spectra of (*S* or *R*)-pSi/PCBM, (*S* or *R*)-pSi/ThCBM, and (*S* or *R*)-pSi/PCBE films before thermal annealing.

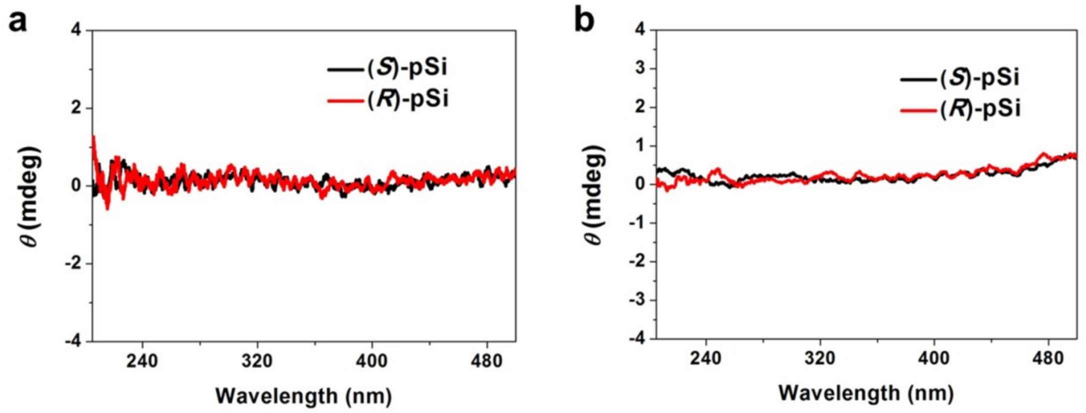

Figure S10. CD spectra of (*S*)-pSi and (*R*)-pSi. CD spectra of (*S*)-pSi and (*R*)-pSi (a) in $CHCl_3$ solutions and (b) in pure films after annealing at 150 °C.

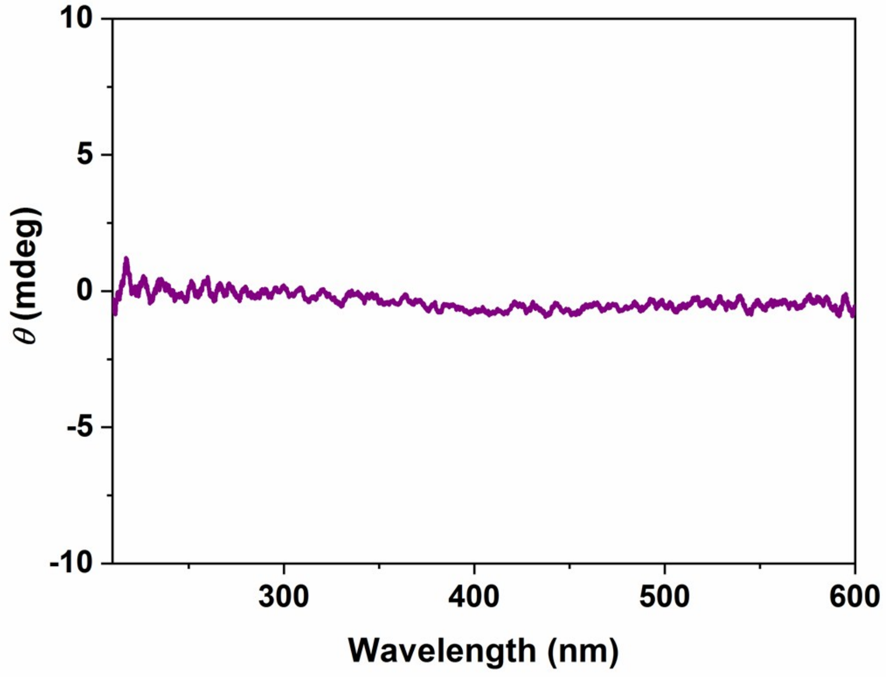

Figure S11. CD spectrum of PCBM film with SSM of a 1:1 mixture of (*S*)-pSi and (*R*)-pSi. The film was prepared by spin-coating the blend solution of (*S*)-pSi (0.75 mg mL^{-1}), (*R*)-pSi (0.75 mg mL^{-1}) and PCBM (10 mg mL^{-1}). After spin-coating, the film was annealed at 150 °C for 30 min.

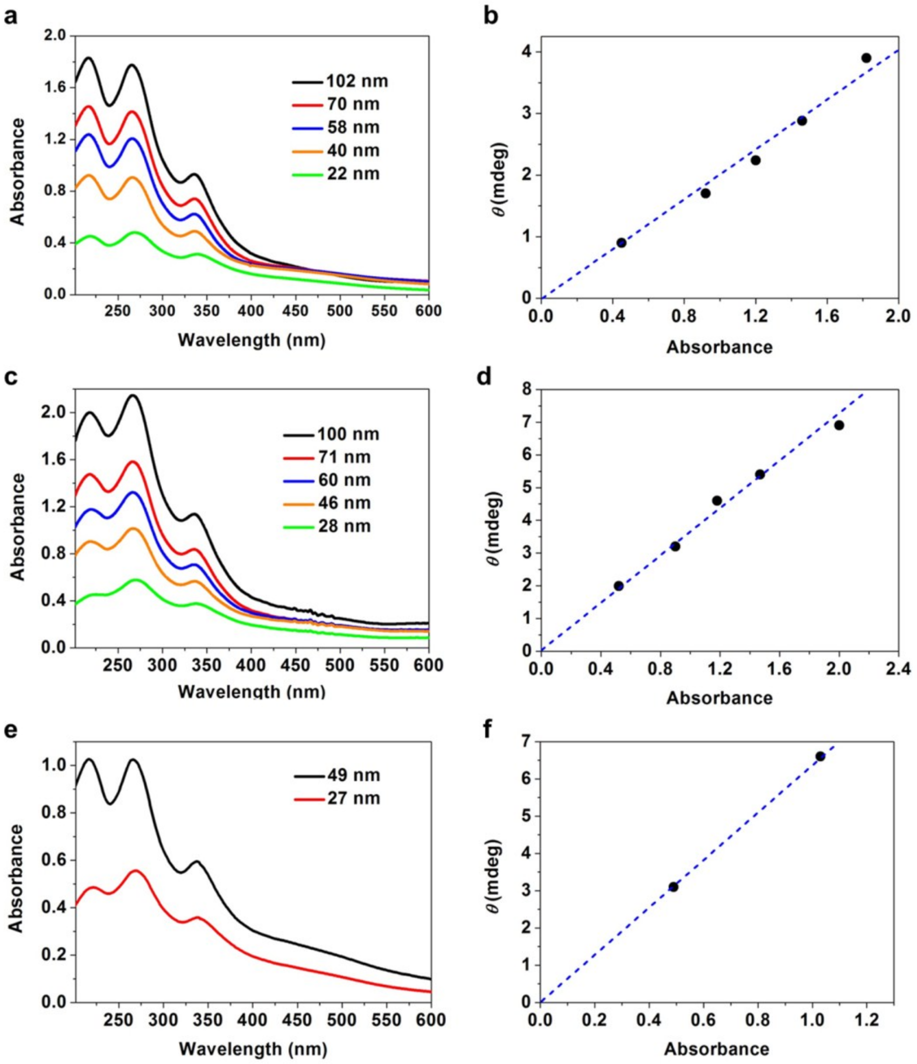

Figure S12. UV-Vis spectra and absorbance dependence of ellipticity. UV-Vis spectra of (a) (*S*)-pSi/PCBM, (c) (*S*)-pSi/ThCBM, and (e) (*S*)-pSi/PCBE films with different thickness values. Ellipticity of the peak at 230 nm in the CD spectra of (b) (*S*)-pSi/PCBM, (d) (*S*)-pSi/ThCBM, and (f) (*S*)-pSi/PCBE films plotted as a function of the absorbance of the UV-Vis peak at around 220 nm.

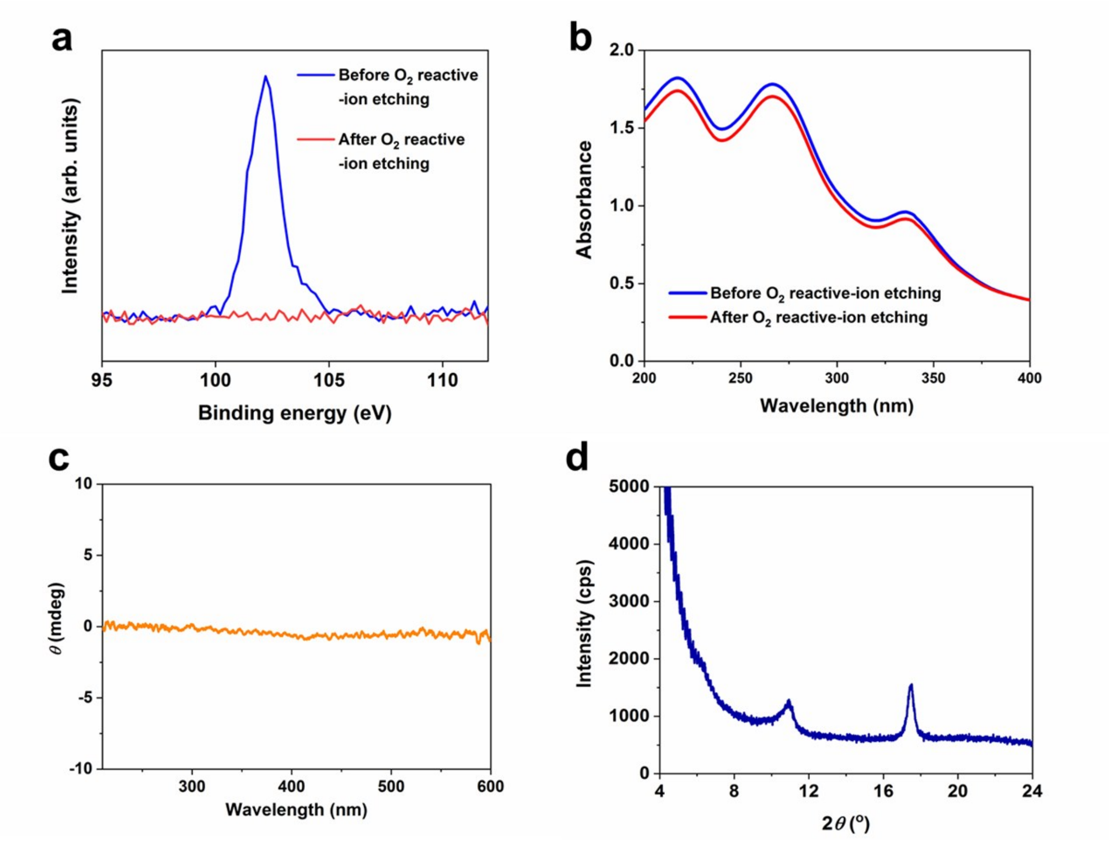

Figure S13. (a) XPS spectra of (*S*)-pSi/PCBM film before and after reactive-ion etching with O_2 plasma for 20 s. O_2 flow rate: 200 mL/min. Si 2p peak disappeared after etching, indicating the SSM of (*S*)-pSi was completely removed. (b) Absorption spectra of (*S*)-pSi/PCBM film before and after oxygen reactive-ion etching for 20 s. The decrease of absorbance after etching is about 4%, suggesting that the thickness of the film was approximately reduced from 100 nm to 96 nm. (c) CD spectra of the film after removing the surface layer from (*S*)-pSi/PCBM film and subsequent thermal annealing at 150 °C for 30 min. No CD signal was observed. (d) Out-of-plane XRD pattern of the film after removing the surface layer from (*S*)-pSi/PCBM film and subsequent thermal annealing at 150 °C for 30 min. The XRD pattern is the same as that of pure PCBM film after annealing, indicating no SSM-induced crystal structure was formed. The (*S*)-pSi/PCBM film in this experiment was prepared by spin-coating the blend solution of (*S*)-pSi (1.5 mg mL^{-1}) and PCBM (20 mg mL^{-1}). The film thickness is approximately 100 nm.

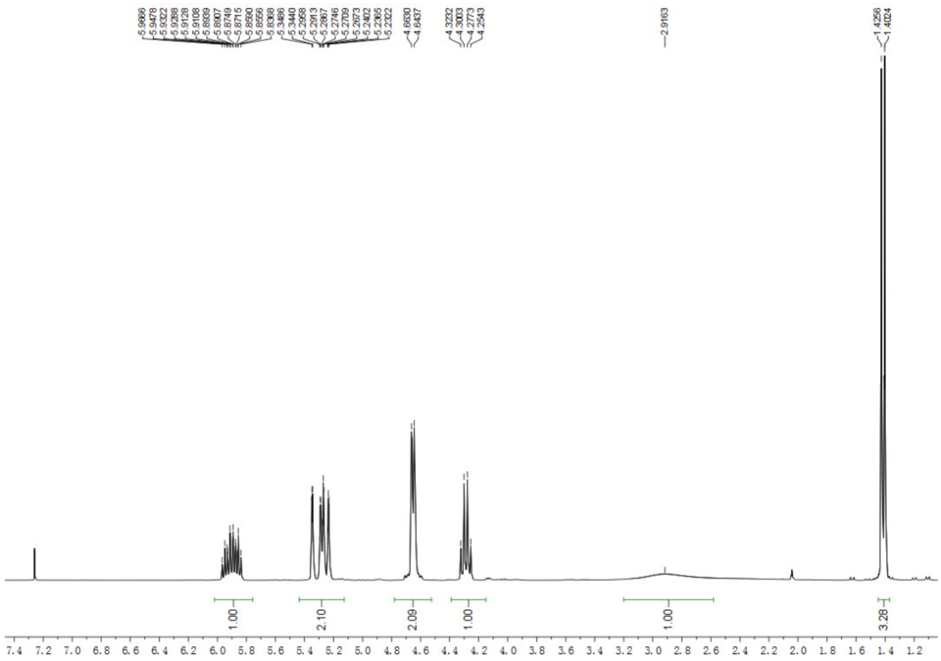

Figure S14. ^{1}H NMR spectrum of **allyl L-(−)-lactate**.

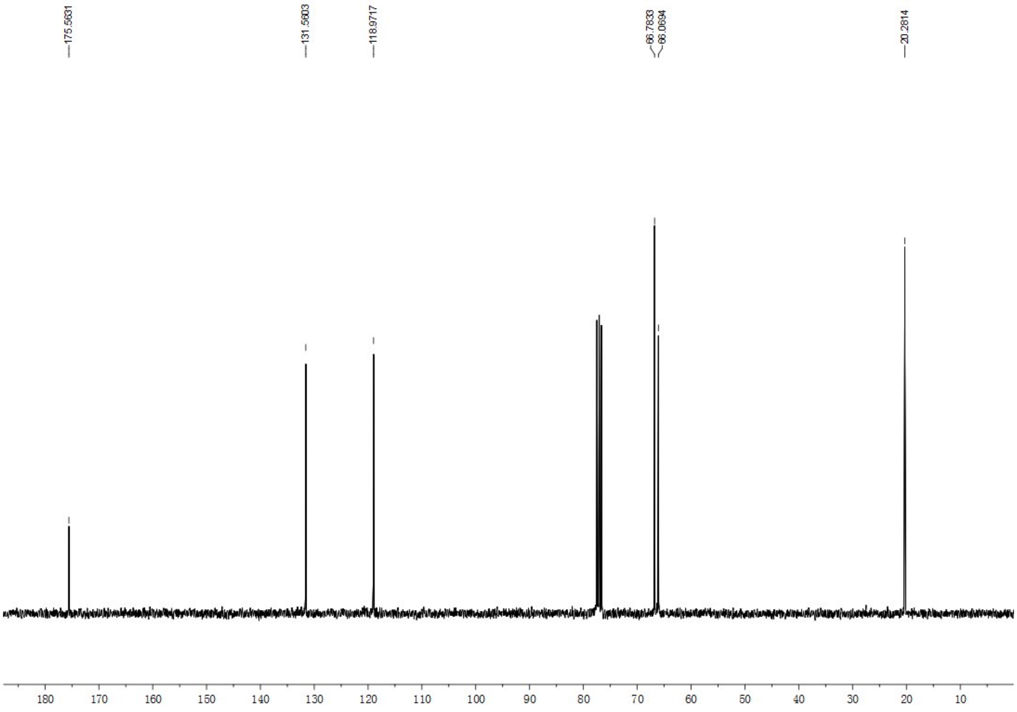

Figure S15. ^{13}C NMR spectrum of **allyl L-(−)-lactate**.

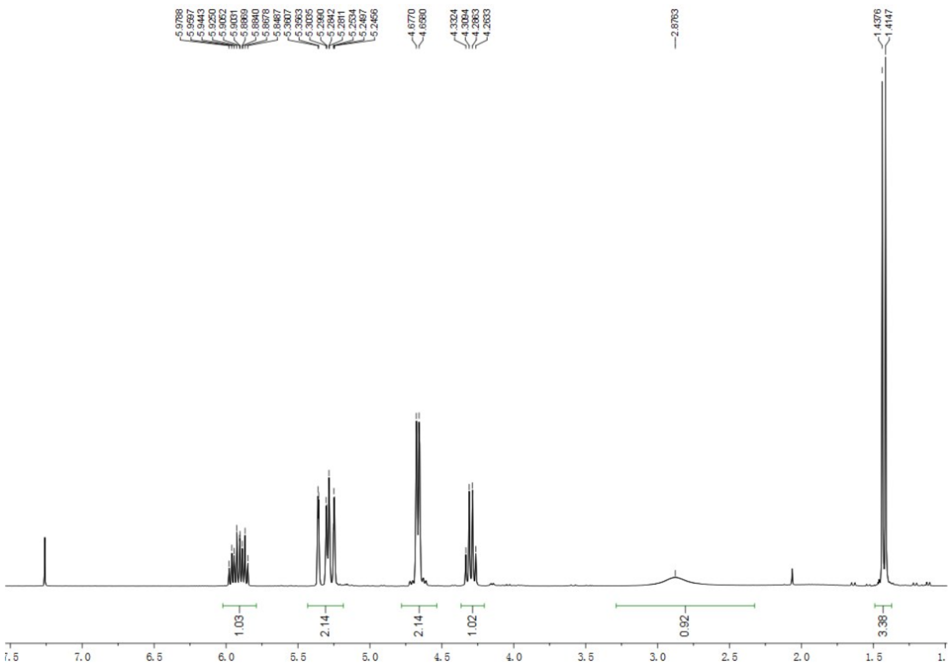

Figure S16. ^{1}H NMR spectrum of **allyl D-(+)-lactate**.

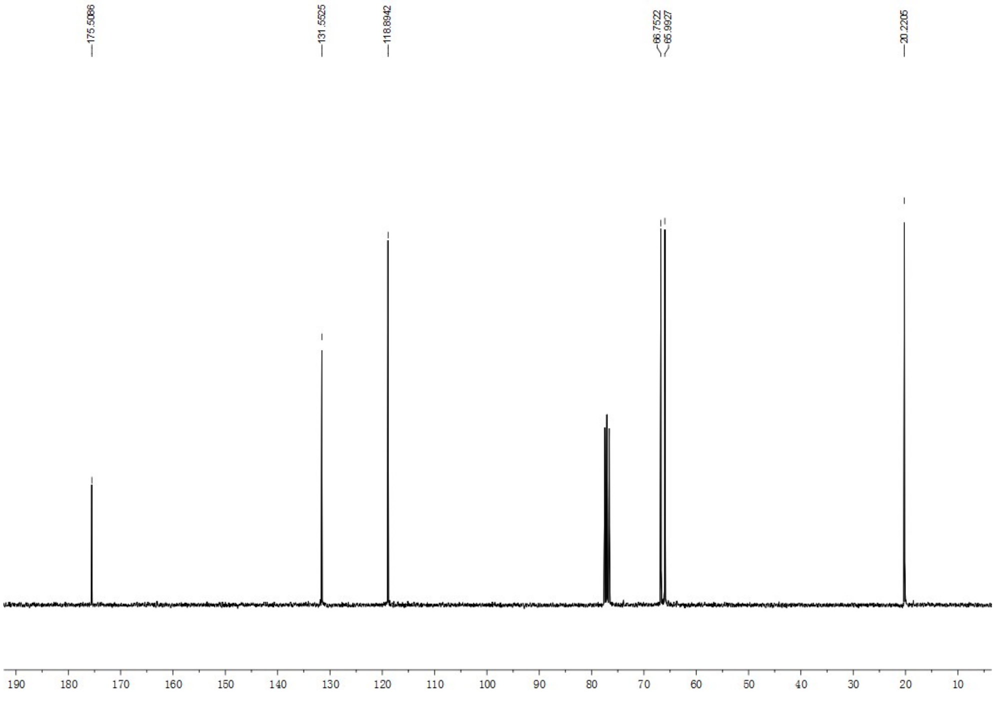

Figure S17. ^{13}C NMR spectrum of **allyl D-(+)-lactate**.

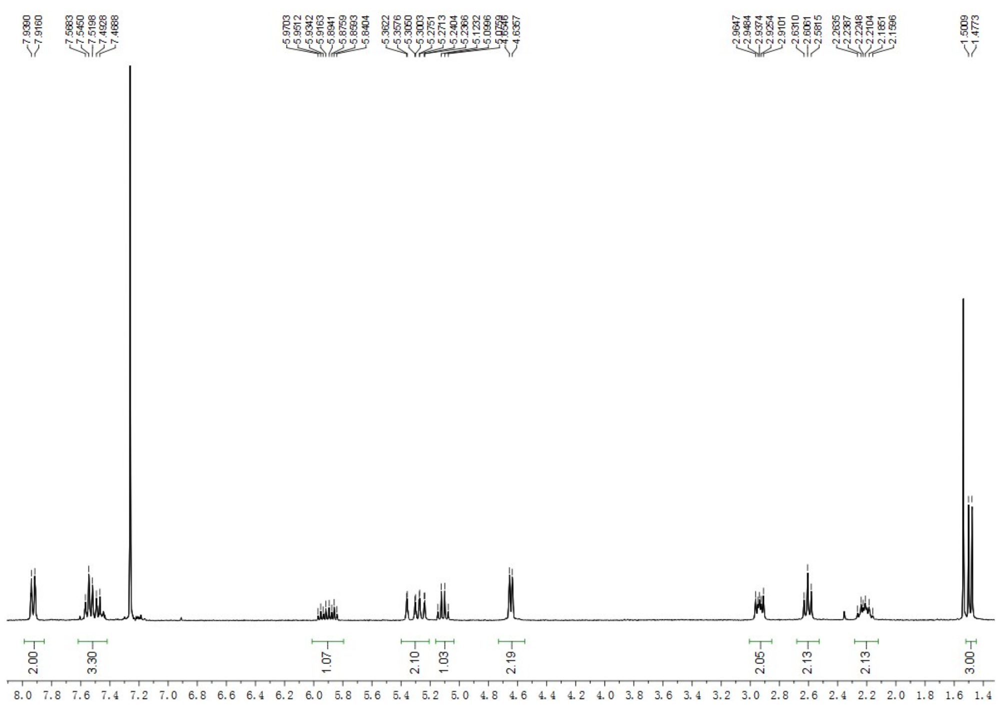

Figure S18. ^{1}H NMR spectrum of **(*S*)-PCB-LA**.

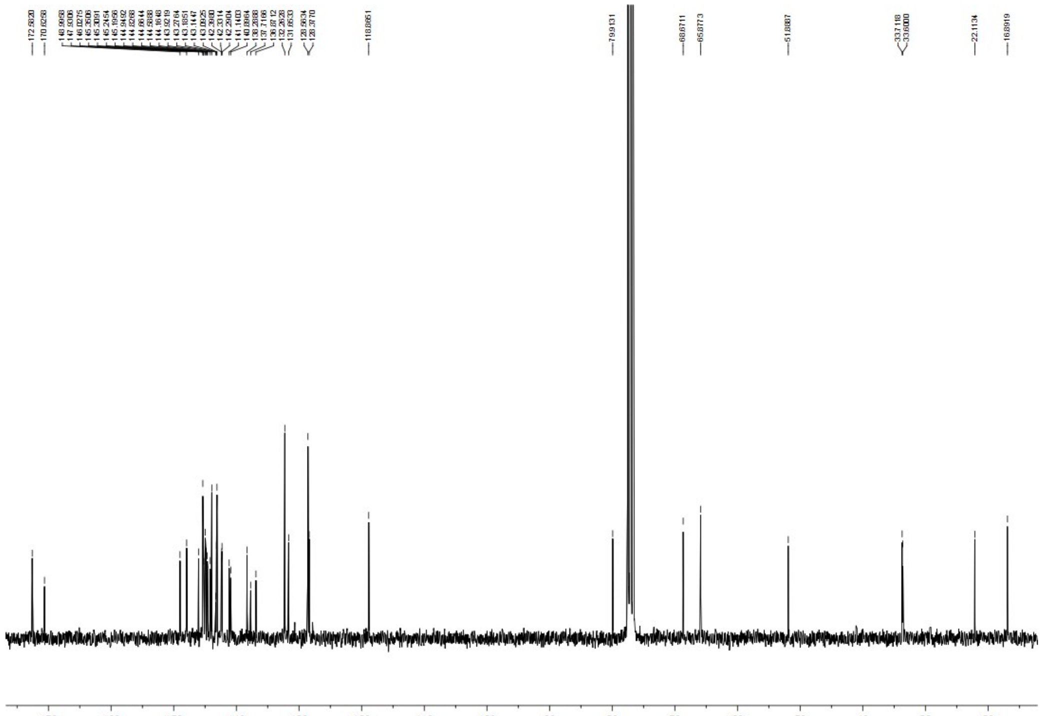

Figure S19. ^{13}C NMR spectrum of **(*S*)-PCB-LA**.

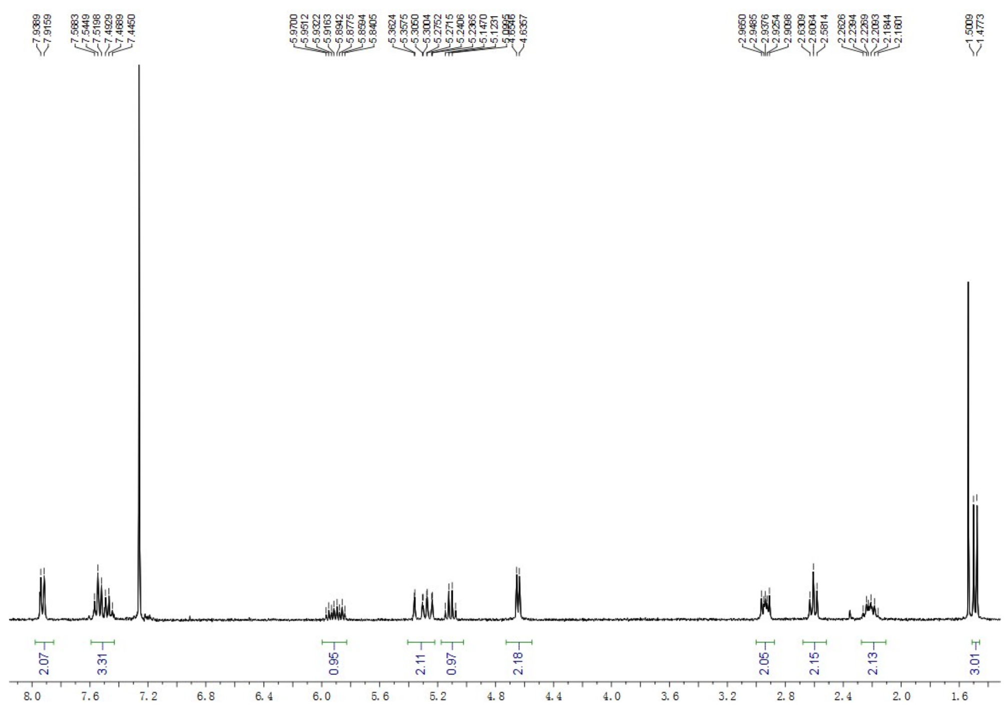

Figure S20. ^{1}H NMR spectrum of ***(R)*-PCB-LA**.

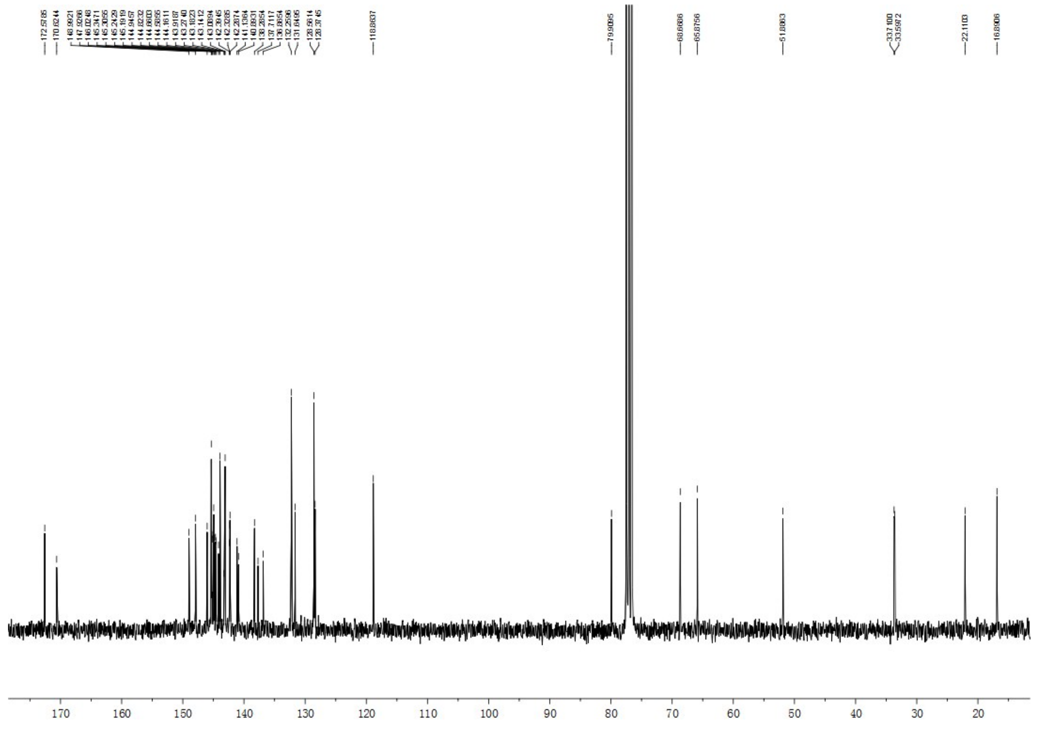

Figure S21. ^{13}C NMR spectrum of ***(R)*-PCB-LA**.

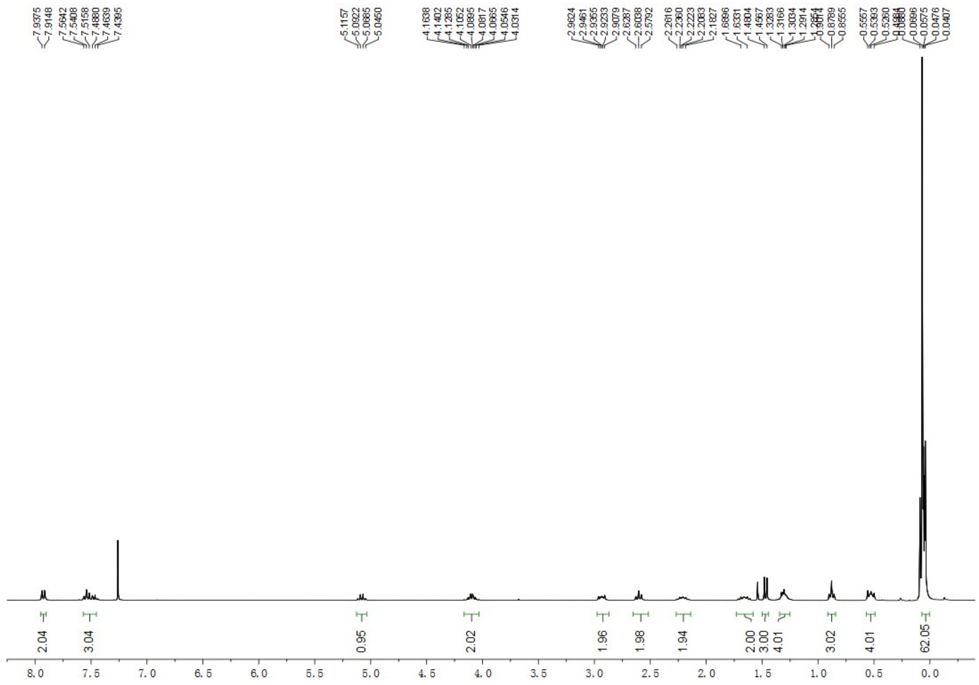

Figure S22. ^{1}H NMR spectrum of **(*S*)-pSi**.

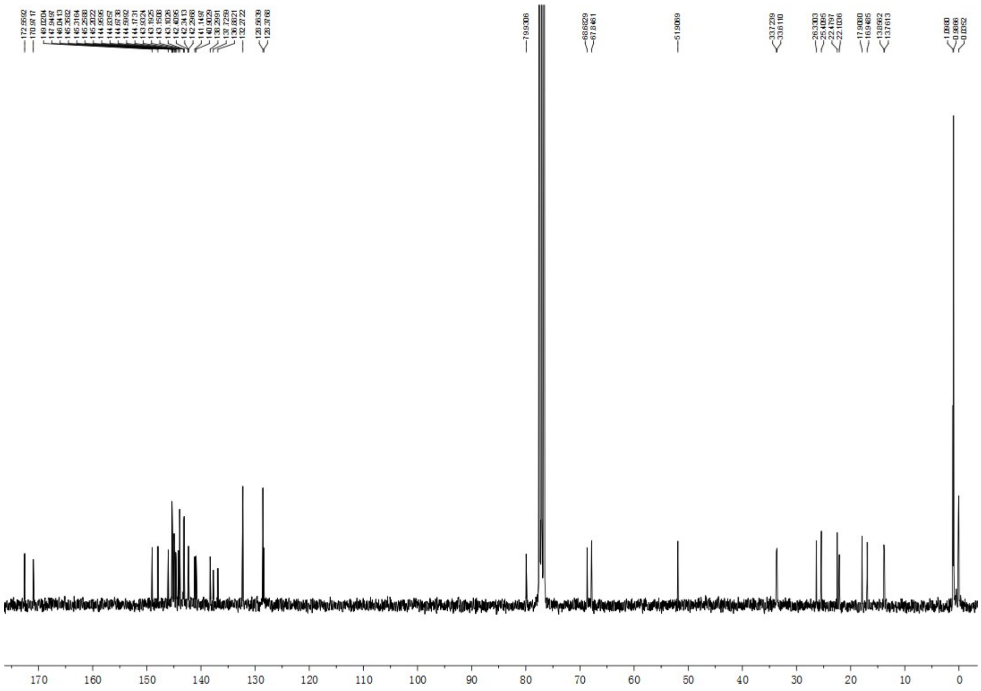

Figure S23. ^{13}C NMR spectrum of **(*S*)-pSi**.

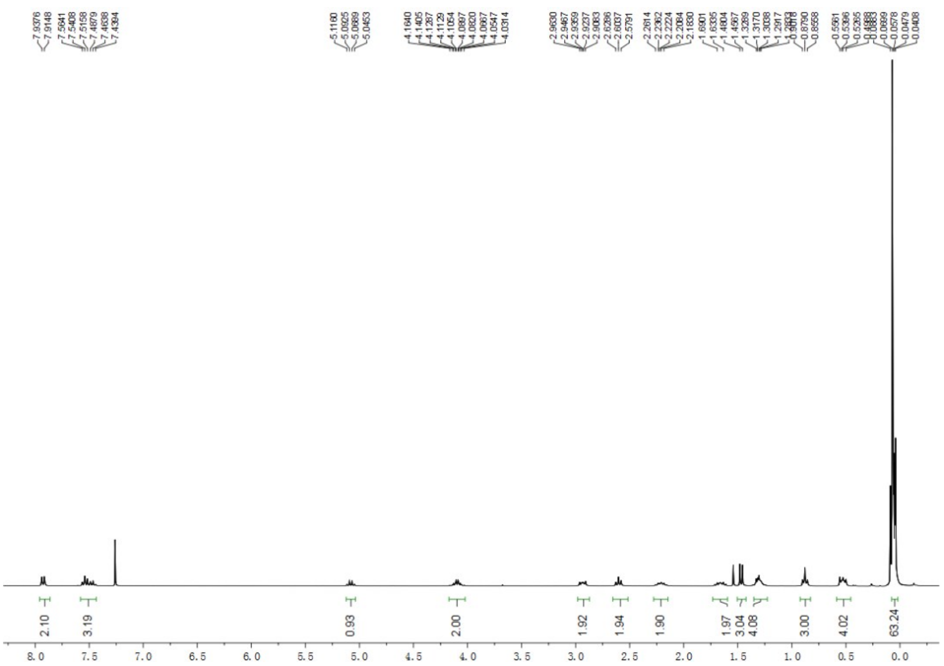

Figure S24. ^{1}H NMR spectrum of ***(R)*-pSi**.

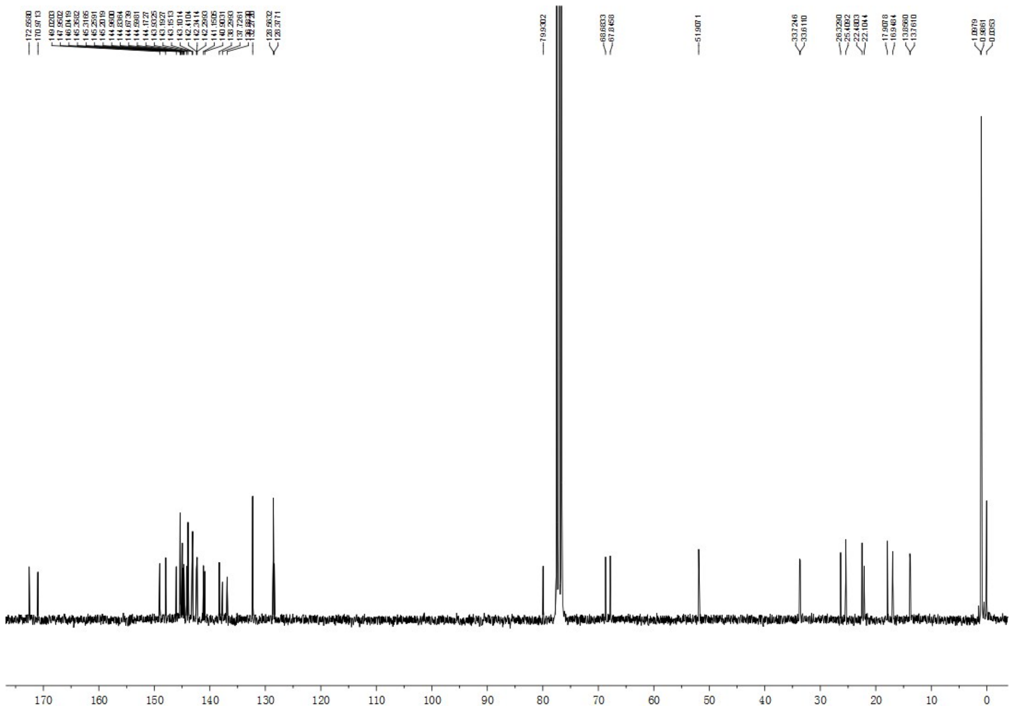

Figure S25. ^{13}C NMR spectrum of ***(R)*-pSi**.

Reference

[1]. Q. Bao, X. Liu, S. Braun, M. Fahlman, *Adv. Energy. Mater.* 2014, **4**, 1301272.

[2]. J. M. Lownsbury, J. C. Sharp, E. J. Mann, C. T. Campbell, *J. Phys. Chem. C* 2015, **119**, 18444-18451.

[3]. C. Ton-That, A. G. Shard, R. H. Bradley, *Langmuir* 2000, **16**, 2281-2284.

[4]. S. Izawa, K. Nakano, K. Suzuki, Y. Chen, T. Kikitsu, D. Hashizume, T. Koganezawa, T.-Q. Nguyen, K.Tajima, *Sci. Rep.* 2018, **8**, 481.
